# Systematic analysis of genome-wide fitness data in yeast reveals novel gene function and drug action

**DOI:** 10.1186/gb-2010-11-3-r30

**Published:** 2010-03-12

**Authors:** Maureen E Hillenmeyer, Elke Ericson, Ronald W Davis, Corey Nislow, Daphne Koller, Guri Giaever

**Affiliations:** 1 Biomedical Informatics, 251 Campus Drive, MSOB, x-215, Stanford University, Stanford, CA 94305, USA; 2 Stanford Genome Technology Center, 855 California Avenue, Palo Alto, CA 94304, USA; 3 Department of Pharmaceutical Sciences, 144 College Street, University of Toronto, Toronto, Ontario, M5S3M2, Canada; 4 Department of Molecular Genetics, 1 King's College Circle, University of Toronto, Toronto, Ontario M5S1A8, Canada; 5 Department of Biochemistry, Beckman Center B400, 279 W. Campus Drive, Stanford University, Stanford, CA 94305, USA; 6 Banting and Best Department of Medical Research, 112 College Street, University of Toronto, Toronto, Ontario MSG1L6, Canada; 7 Department of Computer Science, 353 Serra Mall, Stanford University, Stanford, CA 94305, USA

## Abstract

We systematically analyzed the relationships between gene fitness profiles (co-fitness) and drug inhibition profiles (co-inhibition) from several hundred chemogenomic screens in yeast. Co-fitness predicted gene functions distinct from those derived from other assays and identified conditionally dependent protein complexes. Co-inhibitory compounds were weakly correlated by structure and therapeutic class. We developed an algorithm predicting protein targets of chemical compounds and verified its accuracy with experimental testing. Fitness data provide a novel, systems-level perspective on the cell.

## Abstract 2

The relationship between co-fitness and co-inhibition of genes in chemicogenomic yeast screens provides insights into gene function and drug target prediction.

## Background

Yeast competitive fitness data constitute a unique, genome-wide assay of the cellular response to environmental and chemical perturbations [1-8]. Here, we systematically analyzed the largest fitness dataset available, comprising measurements of the growth rates of barcoded, pooled deletion strains in the presence of over 400 unique perturbations [1] and show that the dataset reveals novel aspects of cellular physiology and provides a valuable resource for systems biology. In the haploinsufficiency profiling (HIP) assay consisting of all 6,000 heterozygous deletions (where one copy of each gene is deleted), most strains (97%) grow at the rate of wild type [9] when assayed in parallel. In the presence of a drug, the strain deleted for the drug target is specifically sensitized (as measured by a decrease in growth rate) as a result of a further decrease in 'functional' gene dosage by the drug binding to the target protein. In this way, fitness data allow identification of the potential drug target [3,4,10]. In the homozygous profiling (HOP) assay (applied to non-essential genes), both copies of the gene are deleted in a diploid strain to produce a complete loss-of-function allele. This assay identifies genes required for growth in the presence of compound, often identifying functions that buffer the drug target pathway [5-8].

The field of functional genomics aims to predict gene functions using high-throughput datasets that interrogate functional genetic relationships. To address the value of fitness data as a resource for functional genomics, we asked how well co-fitness (correlated growth of gene deletion strains in compounds) predicts gene function compared to other large-scale datasets, including co-expression, protein-protein interactions, and synthetic lethality [11-13]. Interestingly, co-fitness predicts cellular functions not evident in these other datasets. We also investigated the theory that genes are essential because they belong to essential complexes [14,15], and find that conditional essentiality in a given chemical condition is often a property of a protein complex, and we identify several protein complexes that are essential only in certain conditions.

Previous small-scale studies have indicated that drugs that inhibit similar genes (co-inhibition) tend to share chemical structure and mechanism of action in the cell [3]. If this trend holds true on a large scale, then co-inhibition could be used for predicting mechanism of action and would therefore be a useful tool for identifying drug targets or toxicities. Taking advantage of the unprecedented size of our dataset, we were able to perform a systematic assessment of the relationship between chemical structure and drug inhibition profile, an essential first step for using yeast fitness data to predict protein-drug interactions. This analysis revealed that pairs of co-inhibiting compounds tend to be structurally similar and to belong to the same therapeutic class.

With this comprehensive analysis of the chemogenomic fitness assay results, we asked to what degree the assay could systematically predict drug targets [2-4]. Target prediction is an essential but difficult element of drug discovery. Traditionally, predictive methods rely on computationally intensive algorithms that involve molecular 'docking' [16] and require that the three-dimensional structure of the protein target be solved. This requirement greatly constrains the number of targets that can be analyzed. More recently, high-throughput, indirect methods for predicting the protein target of a drug have shown promise. Some approaches search for functional similarities between a new drug and drugs whose targets have been characterized. For example, one such approach [17] looks for similarities in gene expression profiles in response to the drug; whereas another [18] looks for similarities in side effects. These and other related approaches require that a similar drug whose target is known is available for the comparison. These approaches are thus limited in their ability to expand novel target space, whereas the model we develop here is unbiased and not constrained to known targets.

An alternative class of approaches to identify drug targets compares the response to a drug with the response to genetic manipulation, with the assumption being that a drug perturbation should produce a similar response to genetically perturbing its target, that is, the chemical should phenocopy the mutation. For example, one class of methods [19,20] searches for similarity of RNA expression profiles after drug exposure to profiles resulting from a conditional or complete gene deletion. A related approach employs gene-deletion fitness profiling, where the growth profiles of haploid deletion strains in the presence of drug are compared to growth profiles obtained in the presence of a second deletion [5]. These approaches are limited in their ability to interrogate all relevant protein targets, both because of scaling issues and because they do not, in the majority of cases, interrogate essential genes, most of which encode drug targets. Finally, over-expression profiling is an approach to drug target identification that relies on the concept that overexpression of a drug target should confer resistance to a compound [21-23].

Our machine-learning approach aims to predict drug-target interactions in a systematic manner using the compound-induced fitness defect of a heterozygous deletion strain combined with features that exploit the 'wisdom of the crowds' [24]; namely, that similar compounds should inhibit similar targets. We designed this approach such that it would effectively leverage the scale of our assay and the size of the resulting datasets. The result is a predictor that infers drug targets from chemogenomic data, and whose performance is sufficiently robust to suggest hypotheses for experimental testing. While experimental testing of direct binding of predicted targets to drugs is beyond the scope of this paper, we accurately predicted known drug target interactions in cross-validation, and provide genetic evidence to verify two novel compound-target predictions: nocodazole with Exo84 and clozapine with Cox17. These results suggest that chemogenomic profiling, combined with machine learning, can be an effective means to prioritize drug target interactions for further study.

## Results

### Co-fitness of related genes

We previously showed that strains deleted for genes of similar function tend to cluster together [1]. Here we greatly expand upon that analysis, quantify the degree to which co-fitness can predict gene function and compare its performance with other high-throughput datasets. To generate a suitable metric, we defined the similarity of gene fitness scores across experiments as a co-fitness value (see Materials and methods). Several measures of co-fitness were tested and we found that Pearson correlation consistently exhibited the best performance in predicting gene function (Supplementary Figure 1 in Additional file 1). Notably, converting the continuous values to ranks or discrete values decreased performance, suggesting that even subtle differences in phenotypic response contain valuable information regarding gene function. Accordingly, Pearson correlation was used for all subsequent analyses.

**Figure 1:**
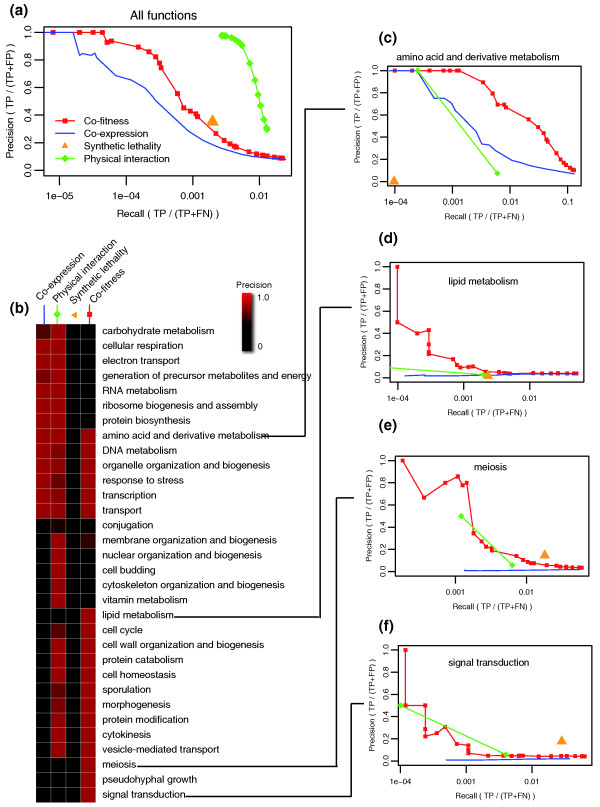
**Predicting shared gene functions using co-fitness and other datasets**. **(a) **Precision-recall curve for each of four high-throughput datasets, illustrating the prediction accuracy of each dataset to expert-curated reference interactions [13]. The optimal dataset has both high precision and high coverage (a point in the upper right corner). TP is the number of true positive interactions captured by the dataset, FP is the number of false positives, and FN is the number of false negatives. Synthetic lethality networks have only one value for precision and coverage because their links are binary. Correlation-based networks, including co-fitness, co-expression, and physical interactions, use an adjustable correlation threshold to define interactions: each point corresponds to one threshold. **(b) **Each cell in the matrix summarizes the precision that each dataset achieved for each function, ranging from low (black) to high (red), hierarchically clustered on both axes. **(c-f) **Individual precision-recall curves for four of the gene categories, from which the values for (b) were calculated. The remaining 28 categories are shown in Supplementary Figure 2 in Additional file 1 in Additional file 1.

We calculated co-fitness separately for the heterozygous and homozygous datasets and evaluated the extent to which co-fitness predicted an expert-curated set of protein pairs that share cellular function, which we refer to as the 'reference network' [13]. Functional prediction performance was compared using several types of functional yeast assays: co-fitness; a unified protein-protein interaction network [25] derived from two large-scale affinity precipitation studies [26,27]; synthetic lethality [28]; and co-expression over three microarray gene expression studies [29-31]. For each of the datasets, we compared the reference network to the predicted gene-gene interactions, at a range of correlation cutoffs for continuous scores (Figure 1a).

We divided our reference network into 32 sub-networks according to the 32 GO Slim biological processes [13]. Each gene pair was assigned to the sub-network if both genes were annotated to that process. The function-specific predictive value of using these sub-networks was assessed using the area under the precision-coverage curve (Figure 1b). The different datasets predicted distinct processes. In particular, co-fitness provided good predictions (relative to other datasets) for functions including amino acid and lipid metabolism, meiosis, and signal transduction (Figure 1c-f; Supplementary Figure 2 in Additional file 1). This observation suggests the chemogenomic assay probes a distinct portion of 'functional space' compared to the other datasets. In other functional categories co-fitness performed less well in its ability to predict gene function. These functions include, most notably, ribosome biogenesis, cellular respiration and carbohydrate metabolism (Supplementary Figure 2 in Additional file 1). Regardless of the underlying reasons why co-fitness performs better for certain functions, this metric clearly provides distinct information that, when integrated with diverse data sources, will aid the development of tools designed to predict gene function [11,12]. Co-fitness interactions are available for visualization [32] and download [33].

The preceding analysis demonstrates that co-fit genes share function. Thus, co-fitness can be used to evaluate the extent to which certain types of gene pairs share function. In an initial test we found that paralogous (duplicated) gene pairs [34] tend to exhibit higher-than-average co-fitness values (*t*-test *P *< 0.01; Supplementary Figure 3 in Additional file 1). This observation argues against a strict redundancy of duplicated genes because if such genes were fully buffered, they would not be expected to exhibit a growth phenotype. Consistent with other recent studies [35,36], our finding supports models that posit that such genes are partially redundant, with deletion of either duplicate resulting in a similar (that is, co-fit) phenotype. Notably, analysis of sequence similarity suggests that paralog co-fitness is not correlated with degree of homology (Supplementary Figure 4 in Additional file 1).

We also found that essential genes were co-fit with other essential genes more frequently than expected. On average, 40% of an essential gene's significantly co-fit partners were also essential genes, compared to only 23% for non-essential gene's co-fit partners (*P *< 6e-45; Supplementary Figure 5a, b in Additional file 1). This observation is consistent with a recent analysis that suggests essential genes tend to work together in 'essential processes' [37,38]. As expected, pairs of co-complexed genes (genes encoding subunits of a protein complex) also exhibit increased co-fitness with other members of the complex (see Materials and methods; Supplementary Figure 5c, d in Additional file 1). Recent analyses [14,15] show that proteins that are essential in rich medium tend to cluster into complexes, suggesting that essentiality is, to a large extent, a property of the entire complex. Indeed, if we define a complex as essential if >80% of its members are essential, 68 of 312 complexes are essential in rich medium, which is significantly greater than that expected by chance [14]. Using our HOP assay (of non-essential diploid deletion strains), we extended this analysis to ask which nonessential proteins might be essential for optimal growth in conditions other than rich media. Using similar criteria (80% of a complex's members are significantly sensitive in a condition), we identified between 0 and 36 conditionally essential complexes over multiple conditions. Overall, 40% of the tested conditions exhibited significantly more essential complexes than were observed in random permutations (*P *< 1e-4), suggesting that condition-specific complexes are pervasive (Supplementary Figure 6 in Additional file 1). For example, in cisplatin (a DNA damaging agent), we observed essential complexes containing Nucleotide-excision repair factor 1, Nucleotide-excision repair factor 2, and other DNA-repair complexes. In rapamycin, the TORC1 complex (a known target of rapamycin) was essential. Several of the other conditionally essential complexes are localized to particular cellular structures, such as the mitochondria and ribosome. Still other condition-specific complexes function in vesicle transport and transcription. For example, in wiskostatin, FK506, rapamycin, and bleomycin, most of the conditionally essential complexes function in vesicle transport. Indeed, vesicle transport genes involved in complexes are, in general, sensitive to a large number of diverse compounds, suggesting that these complexes are required for the cellular response to chemical stress. This finding supports and extends our previous finding that many individual genes are involved in multi-drug resistance [1].

### Co-inhibition reflects structure and therapeutic class

To better understand how a compound's structure and therapeutic mechanism correlates with its effect on yeast fitness, we asked how well compound structure and therapeutic action correlate with the corresponding inhibition profile. For this analysis, we define co-inhibition for a compound pair as the Pearson correlation of the chemical response across all gene deletion strains. Structural similarity was defined as described in the Materials and methods, and therapeutic use was defined using the World Health Organization's (WHO) classification of drug uses [39].

**Figure 2:**
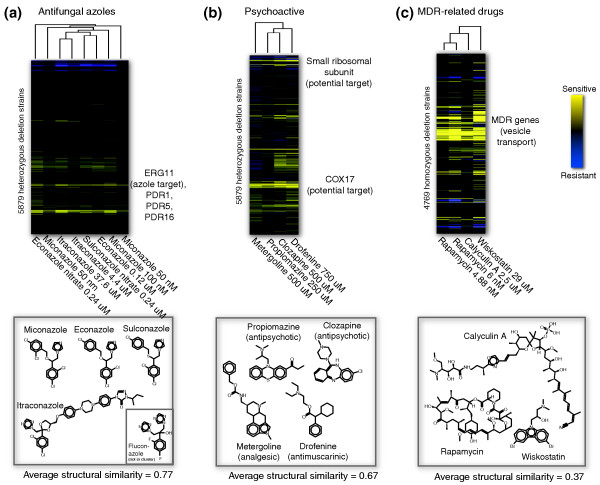
**Compound clusters, extracted from genome-wide two-way clustering on the complete dataset (using all genes and all compounds)**. **(a) **Antifungal azoles in the heterozygous data, with high structural similarity. All induce sensitivity in strains deleted for *ERG11*, an azole target, and related pleiotropic drug resistance (PDR) transport-related genes; fluconazole (inset) did not appear in this cluster, though it is also thought to target Erg11. **(b) **Psychoactive compounds that target dopamine, serotonin, and acetylcholine receptors in human; these compounds cluster in the heterozygous dataset based on inhibition of small ribosomal subunit genes and Cox17, potential targets in both yeast and human. **(c) **Examples of drugs with similar homozygous fitness profiles; the similarity is due to shared sensitivity of strains deleted for multi-drug resistance (MDR) genes with roles in vesicle-mediated transport.

The results obtained from clustering compounds by co-inhibition are summarized in Figure 2. One cluster in the HIP dataset contained four related antifungals (miconazole, itraconazole, sulconazole, and econazole) that exhibit high structural similarity. Each of these related antifungals induced sensitivity in heterozygous strains deleted for *ERG11*, the known target of these drugs [40]. Other genes required for uncompromised growth in these antifungals include multi-drug resistance genes, such as the drug transporter *PDR5 *(the yeast homolog of human *MDR1*), the lipid transporter *PDR16*, and the transcription factor *PDR1*, which regulates both *PDR5 *and *PDR16 *expression [41]. Interestingly, fluconazole did not cluster with the four other azoles, despite evidence that it also targets Erg11 [40,42]. Fluconazole's chemical structure is similar to other azoles except that fluorine atoms are substituted for chlorine (Figure 2a, inset). Consistent with our observation, an expression-based study also detected differences between fluconazole and these azoles [43]. The azole separation found in our clustering analysis demonstrates that the chemogenomic assay can discriminate similar but not identical compounds.

A second HIP cluster (Figure 2b) comprised psychoactive compounds that are annotated as psycholeptics that target dopamine, serotonin, and acetylcholine receptors but do not share structural similarity. Because their neurological targets do not exist in yeast, the sensitivity we observe is likely a result of these compounds affecting additional cellular targets in yeast [44]; these 'secondary' targets, if conserved, may correspond to additional targets of these compounds in human cells. This observation underscores the point that clusters derived from the heterozygous data can identify compounds with similar therapeutic action despite the absence of the target in yeast. In the homozygous data, several drugs with no obvious structural similarity clustered together (Figure 2c): rapamycin, calyculin A and wiskostatin. The similarity in these profiles resulted from inhibition of strains deleted for genes involved in intracellular transport and multidrug resistance [1].

**Figure 3:**
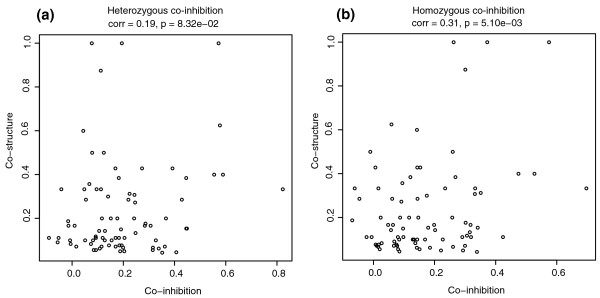
**The limited correlation between Tanimoto structural similarity and co-fitness in the heterozygous and homozygous datasets suggests that chemical structure influences inhibition patterns but does not exclusively define them**. Each point represents a pair of compounds; to allow for comparison between **(a) **heterozygous and **(b) **homozygous datasets, for this figure we used only pairs of compounds that were tested in both datasets.

**Figure 4:**
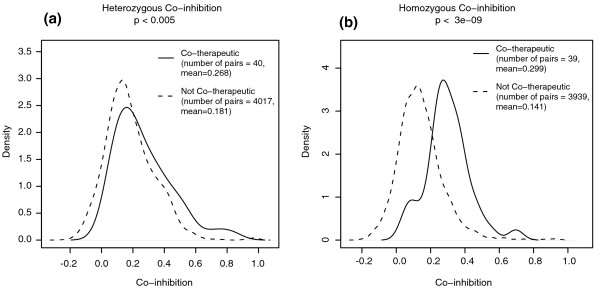
**The ability of co-inhibition to predict shared therapeutic use was higher for the homozygous than for the heterozygous dataset**. As reference, we used a set of compound pairs with shared therapeutic use (WHO ATC level 3 code). As in Figure 3, we used only pairs of compounds that were tested in both the **(a) **heterozygous and **(b) **homozygous datasets.

The clusters highlighted in Figure 2 suggest that co-inhibition can reveal both shared structure and common therapeutic use. We observed a weak correlation between structural similarity and co-inhibition (Figure 3), suggesting that chemical structure may influence patterns of inhibition, but further data on this topic are needed. We note that the compounds used to collect the genome-wide fitness data were chosen to be as diverse as possible; a set of compounds that were more similar would be expected to show a greater correlation between co-inhibition and structural similarity. We also found significant relationships between shared Anatomical Therapeutic Chemical (ATC) therapeutic class [39] and co-fitness profiles, especially for the homozygous dataset (*P *< 3e-9; Figure 4). This finding suggests that a drug's behavior in the yeast chemogenomic assays can be predictive of its therapeutic potential in humans. We noted a correlation between chemical structure and therapeutic class, but a compound's structure alone did not explain the therapeutic relation to co-inhibition. For pairs of compounds that both were positively co-inhibiting (correlation >0) and shared a therapeutic class, more than 70% did not share significant structural similarity (that is, Tanimoto similiarity <0.2). This observation indicates that compounds with very different structures can still produce similar genome-wide effects. This finding can be attributed to structurally diverse compounds that inhibit different proteins within the same pathway, or to different compound structures that inhibit the same target [45,46]. Co-inhibition interactions are available for visualization [32] and download [33].

### Yeast chemical genomic interactions identify drug targets

We extended our observations on the relation between HIP-HOP sensitivities and chemical structure to construct a novel method to address the difficult task of predicting drug targets. Our aim was to use the ensemble of information within the chemical genomic data to better predict the protein target(s) of a compound, and to distinguish which of the sensitive strains is the most likely drug target. We developed a novel machine learning approach to estimate an 'interaction score' between compound *c *and gene *g*. Based on our original observation that heterozygous deletion strains of the drug target are often sensitive to the drug [2-4], we set as a key feature in our model the fitness defect score of heterozygous strain deleted for gene *g *in the presence of compound *c*. Using the fitness defect in isolation, however, ignores potentially useful knowledge about the properties of compound-target interactions. We therefore added several additional features described below (see also Materials and methods).

First, to avoid false predictions involving promiscuous compounds or genes, we included the frequency of significant fitness defects for the gene or compound across the dataset. Second, because structurally similar compounds often inhibit the same target (as in the case of Erg11 in Figure 2a), we constructed features designed to exploit this 'wisdom of the crowds' [24]. Specifically, in predicting the interaction between *c *and *g*, we included features that quantify the structural similarity of a set of compounds that inhibit *g*. For example, in Figure 2a, the average structural similarity (Tanimoto) of four compounds predicted to bind Erg11 was 0.77, a feature that we hypothesized would help identify true interactions. Because co-inhibiting compounds may share targets, we also included features representing the target *g*'s fitness defects relative to *c*'s top ten co-inhibiting compounds.

One challenge in developing this approach was the limited amount of available high-quality data relating to drug targets in yeast. We collected two high-quality training sets: an expert-curated set of 83 yeast protein-compound interactions, and yeast homologs of 180 human drug-protein pairs annotated as interacting in DrugBank [47] (see Materials and methods). We constructed random negative interaction sets in two ways: balanced (equal number of positive and negative examples), and unbalanced (incorporating all possible negative interactions) (see Materials and methods). With these known drug-target interactions and features, we tested several algorithms using cross-validation. Here the algorithm is trained on one portion of the known drug-target interactions, and tested on a held-out (unseen) portion of the known drug-target interactions. We first tested a simple decision stump algorithm, where the model chooses a single feature by which to classify the test interactions. Fitness defect was found to be the most informative feature. We next tested a variety of other algorithms (Supplementary Figure 7 in Additional file 1) on both the balanced and unbalanced training sets. Richer models (such as random forest, logistic regression, and naïve Bayes) that incorporated all features out-performed the simple decision stump model in both the balanced and unbalanced regimes, highlighting the importance of including multiple features. Of the tested algorithms, the random forest algorithm typically yielded the best performance (Supplementary Figure 7 in Additional file 1). This algorithm builds several decision trees and selects the mode of the outputs (see Materials and methods). We compared four models: a simple threshold (decision stump) using fitness defect alone, a random forest using fitness defect alone, a random forest using only the chemical structure similarity features, and a random forest using all features.

**Figure 5:**
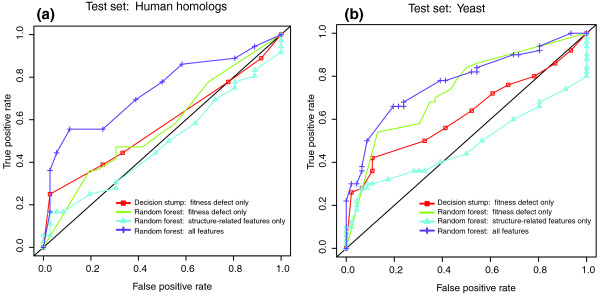
**Drug target prediction accuracy (ten-fold cross validation) using one of four algorithms: log_2 _ratio fitness defect with a simple decision stump model (red); log_2 _ratio fitness defect with a richer random forest model (green); the chemical structure similarity features with the random forest model (blue); and all features with the random forest model (purple)**. Each point represents a threshold for the algorithm. For the decision stump, each point represents a single log_2 _ratio value, and for the random forest, each point represents the algorithm's decision as a mode of decision trees that use the available features (see Materials and methods). The accuracies of other algorithms are shown in Supplementary Figure 7 in Additional file 1. **(a) **Performance on the expert-curated reference set of compounds and their known interacting yeast proteins. **(b) **Performance on DrugBank protein-compound interactions (mostly human) mapped to yeast through protein homology.

The random forest using fitness defect alone performed considerably better than the decision stump (Figure 5), showing that the relationship between fitness defect and compound-target interaction is more complex than a single threshold. Introducing the additional features described above (such as compound structure similarity) gave another considerable boost in performance, particularly in the more challenging dataset of the human homologs from DrugBank (Figure 5a). To quantify the improvement derived from including the other features, we removed features one at a time and re-analyzed the prediction performance (Supplementary Figures 8 and 9 in Additional file 1). Although fitness defect was the most valuable feature, all other features also contributed to the improved performance. Particularly valuable were features that measured shared chemical structure of co-inhibiting ligands, and the median fitness defect of co-inhibiting ligands. However, using chemical structure features alone yielded fairly poor performance (Figure 5). These observations illustrate the usefulness of aggregating information across our genome-wide assay.

The predictive accuracy of our algorithm is of sufficient quality to derive new candidate drug targets for experimental testing. Intuitively, if the protein is a *bona fide *target of the compound, decreasing gene dosage should increase sensitivity to compound (as in the HIP assay) by decreasing the amount of target protein, and increasing gene dosage should increase resistance to compound through overexpression of the target protein [21]. To genetically validate our algorithm's novel computational predictions, we asked if the putative target identified in the HIP assay (decreased gene dosage) confers resistance to compound when overexpressed. It is important to appreciate that the requirements to achieve overexpression rescue are quite stringent. First, the fitness defect induced by compound must be measurable, but cannot be so severe that cells cannot be restored to wild-type growth - that is, the compound must induce a modest but reproducible fitness defect. Second, the 'rescuing protein' must be expressed at a level that can override the compound effect, but not expressed to a level that will inhibit yeast growth [48], which would therefore confound the detection of growth rescue. Accordingly, these experiments may have a high rate of false negatives, but when a specific rescue event is observed, it is likely to be informative. This rationale has been used with success in a study of 188 compounds [23].

**Figure 6:**
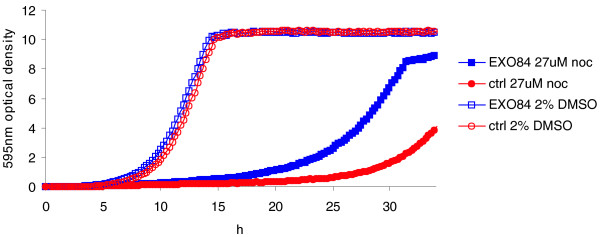
**Overexpression of Exo84 alleviates the sensitivity of the control to 27 μM nocodazole**. The optical density at 595 nm over time for wild-type BY4743 cells harboring the Exo84 overexpression construct compared to that of controls (ctrl) transformed with plasmid lacking a gene insert (for details, see Materials and methods, and for replicates, see Supplementary Figure 10 in Additional file 1).

We tested 4 of our top 12 novel predictions (see Materials and methods; Supplementary Table 1 in Additional file 1) and found pronounced gene-specific rescue of the compound-induced growth defect in two cases. In the first case, we tested our prediction that Exo84 is a target of the microtubule-depolymerizing drug nocodazole using the overexpression approach. We found that overexpression of Exo84 does indeed confer resistance in the presence of nocodazole (Figure 6). The overexpression results were highly reproducible (Supplementary Figure 10 in Additional file 1). In a second experiment, we tested the predicted interaction between clozapine, an FDA-approved drug used primarily to treat schizophrenia, and the yeast protein Cox17. Interestingly, we initially observed robust rescue to clozapine both when yeast and when human Cox17 were overexpressed in yeast, suggesting that human Cox17 may be a target of clozapine (Supplementary Figure 10 in Additional file 1). Subsequent testing of a large number of Cox17 overexpressing clones revealed a more complex pattern: although all overexpression clones conferred resistance, we occasionally observed clozapine resistance in control strains carrying empty vector. The Cox17-independent rescue may be due to the appearance of suppressors in the strain background (data not shown). However, the fact that all overexpression colonies tested showed a pronounced rescue to clozapine when overexpressing Cox17, and loss of Cox17 function (in the HIP assay) conferred sensitivity, strongly suggests an interaction. Detailed biochemical characterization will be required to elucidate the exact nature of this interaction which, based on renewed interest in clozapine [49], is of great medical value.

Two other tested predictions were potential interactions of Pop1 and Arc18 with the drug nystatin. Nystatin is known to bind to membrane ergosterol, and it causes cell death by creating pores in the plasma membrane [50]. For this reason, we did not expect any individual protein, when overexpressed, to be able to rescue this drug-induced defect. However, to avoid biasing our predictions, we tested for nystatin rescue by overexpression of Pop1 and Arc18. As expected, neither protein was able to rescue sensitivity to nystatin when overexpressed.

Combining our overexpression rescue results with those of Hoon *et al*. [23] and others in the literature [23,51,52], we find that 5 of our 12 top compound-target predictions were validated (Supplementary Table 1 in Additional file 1). For the purposes of comparison, Hoon *et al*. [23] found overexpression resistance for only 1% of the compound-gene pairs tested in a competitive growth format, making our validation result highly significant.

## Discussion

Currently, most genome-wide datasets, including expression, protein-protein and synthetic genetic interaction data, have been extensively analyzed to help illuminate cell function. Data continue to be generated, which adds predictive power to these large-scale approaches. In this study, we present the first large-scale, systematic analysis of co-fitness, highlighting its novelty and implications for functional genomics. Specifically, these studies: quantified the ability of co-fitness (the correlation of fitness profiles of all genes across all drugs) to predict the functions of genes not evident in other large-scale assays; quantified the degree to which co-inhibition (the correlation of fitness profiles of all drugs across all genes) correlates with both chemical structure and therapeutic action; and demonstrated that a machine-learning model derived from these data predicts drug-target interactions.

We first showed that, overall, co-fitness data identify gene function better than co-expression data but not as well as the physical interaction dataset when compared to a gold standard [13]. When we examined the predictability for specific functions, co-fitness predicts certain functions much better than other large-scale datasets. These functions (underrepresented in other large-scale datasets) include amino acid and lipid metabolism, meiosis, and signal transduction (Figure 2c-f; Supplementary Figure 2 in Additional file 1). This interesting finding suggests different biological processes are better suited to different genome-wide approaches. The fact that signal transduction is predicted relatively well by co-fitness, for example, may be explained by the fact that signal transduction is often a rapid response occurring on the order of milliseconds, a time frame too short to allow expression and translation of required proteins [53,54]. It is not surprising, therefore, that co-expression performed poorly in this regard. Functions for which co-fitness performed more poorly than either expression or protein-protein interaction data include ribosome biogenesis, cellular respiration and carbohydrate metabolism. This result may be due to a high degree of redundancy of these functions or because these functions are not involved in the response to drug perturbation.

Two other findings arose from the functional analysis. First, duplicated genes were co-fit with their duplicate partners and the degree of co-fitness for this set of genes was independent of their sequence similarity. This finding supports the hypothesis of partial, rather than strict, redundancy [35]. Second, we demonstrated the prevalence of conditionally essential complexes, suggesting that essentiality is often a property of complexes rather than individual genes [37,38].

We also provide a first systematic analysis of co-inhibition, and show that we can identify both structural and therapeutic relationships between compounds. While the correlation of co-inhibition to co-structure was significant, it was not very high. This may be due, in part, to the fact that our library was chosen for maximum diversity. The correlation of co-inhibition to therapeutic use was somewhat surprising because the therapeutic classes of the compounds reflect their human use while the co-inhibition results are based on yeast fitness measurements. The correlation between co-inhibition and therapeutic use might, in fact, be an underestimate because our current analysis is limited by the quality and quantity of the therapeutic data available. Our representations of chemical structure and drug therapeutic use rely on public databases, which will undoubtedly improve over time.

Importantly, we showed that fitness profiling can help to identify the most likely target of a given compound from a candidate group of sensitive yeast deletion strains. Traditional drug discovery efforts often focus on the activity of a purified protein target in isolation. These *in vitro *approaches are useful for maximizing the potency of a given inhibitor, but invariably ignore factors critical for understanding drug action, including cell permeability and the potential interaction/inhibition of other proteins in a cellular context. *In vivo *chemical genomic assays address these limitations, and can provide a more comprehensive view of drug-protein interactions. Such results can play an invaluable role in understanding and predicting a compound's clinical effects and in guiding its use, including predicting secondary, unwanted drug targets. New methods for target identification are of enormous value because the coverage of current methods is limited. Traditional computational approaches to drug-target prediction require three-dimensional structure of the protein to predict binding, often by 'docking' the ligand into the binding pocket of the protein [16,55]. The success of these methods to date has been variable, with some studies able to predict known interactions with significant enrichment, and others performing worse than random [55-57]. These methods are also limited to those proteins that have solved three-dimensional structures. Other computational methods utilize protein sequence rather than chemical structure, but these methods are only applicable to individual proteins or a small subset of proteins that possess a high degree of similarity [58-60]. We compared our results to a sequence-based method, testing our gold standard against the interaction model built by [58], but the model was unable to make predictions about any of these known interactions, presumably due to the lack of sequence similarity to the available training sets.

Thus, new sources of data and accompanying computational methods can be of significant value. Our study of genome-wide fitness experiments suggests that fitness profiling offers a new, complementary approach to generate quantitative, testable predictions of drug target interactions, including predictions that may be outside the scope of previous computational approaches. Using this approach, we predicted both known and novel interactions, and provide independent experimental evidence for two novel interactions. Our algorithm predicted that the Exo84 protein interacts with nocodazole and that the Cox17 protein interacts with clozapine. Genetic gene-dose modulation experiments supported these findings. These genes, when overexpressed, rescued their respective drug-induced fitness defect in wild-type cells, providing independent experimental evidence of a predicted interaction.

The first validated prediction is the interaction of Exo84 with nocodazole. Exo84 is a subunit of the well-conserved exocyst complex, first identified for its role in the secretory pathway in *Saccharomyces cerevisiae *[61]. The mammalian homolog is essential for development and participates in multiple biological processes, including vesicle targeting to the plasma membrane, protein translation, and filopodia extension [62,63]. Filopodia are cytoplasmic projections that extend from the leading edge of migrating cells and are important for cellular motility. Like nocodazole, the exocyst complex inhibits tubulin polymerization *in vitro *[64]. It is known that the microtubule-depolymerizer nocodazole distorts the filamentous localization of Exo84 in cultured mammalian cells [64]. Furthermore, the exocyst localization is dependent on microtubules in normal rat kidney (NRK) cells, and the filamentous distribution of Exo84 (as well as two other exocyst subunits, Sec8 and Exo70) is disrupted by nocodazole. Accordingly, it is possible that in yeast, nocodazole treatment causes mislocalization of Exo84, preventing the protein from performing its essential role in the exocyst.

A second intriguing finding is our prediction of an interaction between clozapine and both yeast Cox17 and its human homolog. Clozapine's primary targets are thought to be neurotransmitter receptors, but the drug also alters cytochome C oxidase (COX) activity through an unknown mechanism [65]. This COX alteration has been shown to be linked to clozapine's side effects [66,67]. Our preliminary genetic data indicating a novel interaction between Cox17 and clozapine are tantalizing given the renewed interest in this drug [61], and deserve further investigation.

Our statistical and experimental results demonstrate the ability of our novel algorithm to produce high-quality, testable hypotheses regarding drug-target interactions. Our model is, however, limited by the number of full-genome chemogenomic profiles obtained and will likely improve as we collect additional data with diverse compounds. Nonetheless, these results may shed new light on the mechanisms by which a drug exerts its primary or secondary effect. Combining this predictive method with other computational and experimental data sources should improve these predictions and expand the number of potential compound-protein pairs for subsequent testing. This technology can easily be implemented in a high-throughput manner and should have a positive impact on the early stages of drug discovery, both by identifying potential new drug targets and as a filter to prune less promising ones.

## Materials and methods

### Data sources

The chemogenomic dataset analyzed here has recently been published [1]. Briefly, it consists of genome-wide fitness data obtained when 5,984 pooled heterozygous and 4,769 pooled homozygous *S. cerevisiae *single deletion strains were grown in 726 and 418 conditions, respectively. The diverse set of compounds tested included FDA-approved compounds, natural products, bioactives, and other chemicals from various compound suppliers [1].

### Co-fitness and co-inhibition

We calculated co-fitness and co-inhibition separately for the heterozygous and homozygous datasets. To determine the best representation of co-fitness of two genes (the similarity of their fitness profiles), we tested several score types and similarity metrics. The score types included log_2 _ratio, z-score, *P*-value, and log *P*-value. Four distance metrics were calculated across all experiments: Pearson correlation; Spearman rank correlation; Euclidean distance; and the cosine of binary data (discretized with a binary cutoff of log_2 _ratio >0.5). Two more distance metrics were calculated across subsets of experiments, based on a biclustering analysis on the log_2 _ratios, using the BicAT toolbox [68] with the Iterative Signature Algorithm [69]. The two metrics were: bicluster co-occurrence count (that is, the number of biclusters in which a gene pair co-occurred); and bicluster Pearson correlation (that is, the correlation in the subset of experiments in the bicluster to which the gene pair was co-assigned; if co-assigned to multiple biclusters, then the correlation was collapsed into the median). We selected Pearson correlation of log_2 _ratio fitness defect scores, which best revealed the reference expert-curated interactions (Supplementary Figure 1 in Additional file 1). In cases where we needed to define a significance cutoff for 'co-fit partners' (Supplementary Figure 5 in Additional file 1), we calculated significant co-fitness (*P *< 0.01, three standard deviations above the mean) as deletion strains with a Pearson correlation coefficient >0.47. Once the Pearson correlation was established as the best metric, to measure the accuracy of predicting gene-gene functional relationships for co-fitness and other gene interaction datasets (Figure 1), we followed the method of [13], using the GRIFn tool [70].

When comparing co-fitness to protein complexes (Supplementary Figures 5 and 6 in Additional file 1), we used three datasets of protein complexes: Munich Information Center for Protein Sequences (MIPS) hand-curated complexes; the TAP-MS-based predicted complexes of Collins *et al*. [25] defined using a score cutoff of 0.5; and Gene Ontology cellular components. We only considered Gene Ontology components with fewer than 25 proteins, excluding the extremely large complexes such as the ribosome. We combined all three datasets, resulting in a large set of 54,255 co-complex interactions.

### Conditionally essential protein complexes

To define conditionally essential protein complexes, we used the 215 expert-curated *Saccharomyces *Genome Database Gene Ontology cellular component complexes with ≥2 members that were viable as homozygous deletions. We limited the analysis to homozygous deletions for simplicity, because complex stoichiometry, which would be affected by a heterozygous deletion, is not sufficiently understood [9]. For each condition, we generated a random distribution by randomly reassigning the genes to the 215 complexes, maintaining complex sizes. We repeated this reassignment 10,000 times. This random distribution allowed us to measure significance in the actual distribution for the condition.

A protein complex was defined to be 'essential' if at least 80% of its genes had a significant (*P *< 0.01) fitness defect. We note that this is slightly different from the original definition of essential genes, whose deletion strains are inviable [71]. Because inviability is difficult to measure in the pooled assay, we chose a *P*-value cutoff instead.

We identified conditions with significantly more essential complexes than random as conditions having ≥*X *essential complexes, where *X *essential complexes were not observed in any of the 10,000 permutations for the condition. This corresponds to a *P*-value <1e-4, and a Bonferroni correction for the 418 conditions tested resulted in a *P*-value of 0.04. *X *was usually around four to five essential complexes, depending on the condition. In order to better rank the hypotheses, we also calculated the average log_2 _ratio fitness defect of each complex in each condition.

### Chemical structure similarity and therapeutic classifications

Because we sought to identify differences between the heterozygous and homozygous datasets in the analysis of shared structure and therapeutic class, we limited the compounds to those tested in both the heterozygous and homozygous assays. This was to allow for fair comparison between the two assays. To analyze chemical structure, we represented each chemical in a machine-readable manner as SMILES (Simplified Molecular Input Line Entry System) strings [72]. We used PerlMol [73] to search each SMILES structure for what we call 'substructure motifs'. Substructure motifs are fingerprint substructures defined by PubChem [74]; we used the 554 SMILES and SMARTS (SMiles ARbitrary Target Specification) substructures (numbered 327 through 880). Some of these substructures are explicit, such as a six-carbon ring with aromatic bonds, while others are more general regular expressions, such as a ring with any type of bond (SMARTS [75]). These substructure motifs range in size from two to eight atoms.

To identify a similarity metric for shared structure, we constructed the substructure vectors (containing the 554 PubChem substructures) in three different ways. First, we used the simple binary vector, where the value is 1 if the compound contains the substructure, and 0 otherwise. Second, we converted each binary indicator in the 554-vector to an inverse document frequency (IDF) score used in text mining [76] to identify infrequent words that may be more informative than common ones. The IDF score for each substructure motif *i *is:

where |C| is the total number of compounds, and c_*j *_is the number of compounds that contain motif m_*i*_. Using IDF limits the analysis to less common, more discriminative substructures. The value for each substructure is 0 if the compound does not contain the substructure, and IDF >0 if it does. Third, we chose a threshold for IDF and converted the IDF vector back into a binary vector, which again is 1 if the compound contains the (relatively infrequent) substructure, and 0 otherwise. Any commonly occurring substructure (with IDF less than the threshold) will always be 0.

For the binary data (that is, the 554-substructure PubChem fingerprints), we calculated structural similarity using the Tanimoto (Jaccard) coefficient, Hamming distance, and Dice coefficient. For the IDF data, we used cosine distance Pearson correlation, Spearman correlation, Euclidean distance, Kendall's Tau, and city-block distance. Although several metrics revealed a relationship to co-inhibition, we found the greatest relationship when we used the IDF vector transformed into a binary vector (where the threshold was IDF >2.5), using the Tanimoto coefficient, which only uses the present substructures (ignoring the 'off' bits). Not surprisingly, this suggests that the less common, more discriminative substructures are more predictive of the compound's activity in this assay.

We defined a pair of compounds to be 'co-therapeutic' if they shared annotation at level 3 of the WHO ATC hierarchy [39]. This level encodes the compound's therapeutic/pharmacological action, such as 'antipsychotics', 'immunosuppressants', and 'antimetabolites'.

In counting the number of co-inhibiting pairs that were co-therapeutic but not co-structural, we first tried limiting the analysis to the pairs tested in common between heterozygous and homozygous datasets, as described for the previous analyses. However, as the sample size in this case was too small to draw conclusions, we expanded this analysis to all compound pairs. We counted pairs of compounds that were positively co-inhibiting (correlation >0), had shared therapeutic class, and a measurable structural similarity (295 and 37 pairs in the heterozygous and homozygous datasets, respectively). Of these pairs, 74% and 90% in the heterozygous and homozygous datasets, respectively, did not share structural similarity (Tanimoto similarity <0.2). We summarized this result in the main text as more than 70% because the two datasets cannot be compared in this analysis.

### Drug target prediction algorithm

To learn a model of protein-compound interactions, we used two curated sets of interactions. First, we asked experts in our laboratory for known protein-ligand interactions in yeast and from this set included those for which the literature provided evidence of the interaction. This yielded 83 expert-curated yeast protein-ligand interactions. We constructed a negative test set as 83 random combinations of compound-protein pairs from the positive test set, forbidding any pairs that existed in the positive set, and keeping constant the connectivity degree of each protein and compound. Second, we used known compound-protein interactions in DrugBank [47] and mapped them to yeast homologs using BLASTp [77] with an e-value <1e-10 and no length criterion, and retained any interactions that both had a yeast homolog and a compound tested in our chemical genomic assay. This yielded a total of 180 unique positive drug-protein interactions. Of these, 134 interactions were due to only two of the DrugBank compounds, staurosporine and emodin, which have many annotated human kinase targets, each of which had up to several BLASTp-determined homologs in yeast. We filtered out these promiscuous compounds, leaving a filtered set of 46 interactions. We again constructed negative interactions (180 unfiltered or 46 filtered) randomly from the proteins and compounds in the positive set. Within each set, we predicted targets using tenfold cross-validation. For DrugBank, the filtered set of 46 interactions showed slightly better performance than the unfiltered set of 180; the filtered set is shown in Figure 5a. In addition to cross-validation on equal-sized positive and negative sets, we also performed cross-validation in which the negative set contained all possible protein-ligand interactions (2,178 and 710, respectively, for the yeast curated interactions and the DrugBank human homologs). These results are shown in Supplementary Figure 7 in Additional file 1.

Some compounds were screened against the deletion collection multiple times, at varying concentrations and time-point of collection. When constructing the training and test sets, a protein-compound pair has multiple possible fitness defect scores, each of which could be considered an individual instance or a replicate to be collapsed with other instances. Here we collapsed such replicates and only considered unique instances of a pair. We used the maximum fitness defect (greatest sensitivity) observed for the strain in the compound.

### Features used in learning drug targets

We defined the following 20 features for use in machine learning for predicting whether a protein-compound pair physically interacts. For each feature we list its name in Supplementary Figures 8 and 9 in Additional file 1.

#### Fitness defect score (two features)

This feature is the heterozygous fitness defect score of the protein-compound pair under consideration (more specifically, the gene deletion-compound pair). We used only the heterozygous (not homozygous) scores because they have been shown to identify the drug target [2,3]. We hereafter denote the gene deletion instance (and its corresponding protein) as *g*_*i*_, and the compound, or drug, under consideration as *c*_*j*_. Fitness defect as a feature uses the original observation, described above, that drug compounds sensitize the heterozygous deletion strain of the physical protein target. For example, the drug methotrexate is known to target the protein Dfr1, and the *dfr1 *heterozygous deletion mutant exhibited a large fitness defect [3]. For this feature, we included both the log-ratio score and *P*-value score ('ratio' and 'pvalue' in Supplementary Figures 8 and 9 in Additional file 1).

#### Gene sensitivity frequency (one feature)

This feature ('genefreq') describes the number of compounds causing sensitivity in *g*_*i *_as a heterozygote. Our motivation for including it is the following: in addition to the physical target, genes involved in bioavailability sometimes cause a significant fitness defect when deleted as heterozygotes. These genes may export the drug from the cell or sequester or metabolize drugs in intracellular compartments such as the vacuole. For example, in the case of methotrexate, we observed significant sensitivity of the strain deleted for *YBT1*, a membrane ABC transporter that expels methotrexate from the cell. In its absence, the cell exhibits sensitivity to methotrexate. We found that these bioavailability gene deletions are frequently sensitive [1]. Such promiscuous strains are less likely to be a specific target, and more likely involved in availability or metabolism.

#### Drug inhibition frequency (one feature)

This feature ('drugfreq') describes the number of heterozygous deletion strains sensitive in *c*_*j*_. Some compounds inhibit large numbers of strains, making it difficult to determine their exact target. For example, some antifungal azole drugs such as miconazole inhibit several hundred strains, only one of which is deleted for the known target *ERG11*, (Figure 2) [1].

#### Phenotype in rich medium (one feature)

In the absence of drug, if the homozygous deletion strain *g*_*i *_initially showed no phenotype relative to wild type [9,78], the target cannot be identified by the heterozygous deletion assay as this assay is dependent on diminished 'functional dosage' of the drug target based on growth inhibition (that is, the heterozygous deletion of the target together with the drug's inhibition by binding to the target protein should mimic a full deletion). The range of phenotypes of homozygous deletions has been measured [9] and can thus serve as a feature ('hompheno').

#### Chemical structure similarity enrichment of putative compounds (three features)

If a gene deletion strain *g*_*i *_is sensitive in the HIP (heterozygous) assay to several structurally similar compounds, this increases confidence that each of the compounds target the protein. Protein-compound binding is often due to a structural backbone on the compound (sometimes called a pharmacophore), which can appear in multiple compounds. If such a common backbone is observed in a set of compounds, it is more likely that the entire set shares a target, compared to a random set of compounds that do not share structure. For example, the protein Erg11 binds to the compounds in Figure 2, which appear structurally similar.

We quantified the structural similarity of a set of compounds as the molecular fingerprints described above. For each gene deletion *g*_*i*_, we considered the set of compounds *C*_*i *_that induced significant sensitivity (*P *< 0.01) in *g*_*i*_. Each individual compound *c *contains a set of motifs, *M*_*c*_. We calculated whether the set *C*_*i *_was statistically enriched for any shared motif *m*, using a log risk ratio (RR), which has been applied in calculating other types of motif enrichment [79]:

where *P*(*m | C*_*i*_) is the frequency of motif *m *among the subset of compounds *C*_*i *_that inhibit *g*_*i*_, and *P*(*m*) is the frequency of motif *m *in all compounds. We also considered pairs of motifs, to allow for the possibility of binding on non-contiguous structural areas of the compound; these were subjected to the same analysis. We chose the motif or pair with the maximum risk ratio score, and used this score as a feature ('struct_enrichment').

As a second type of feature in this realm of structural enrichment ('struct_count'), we simply counted the number of compounds *k *in *C*_*i *_that all shared a common motif with the compound in question. For example, when predicting whether *ERG11 *binds miconazole (Figure 2a), we would observe that three other compounds in *C*_*ERG*11 _share a common motif (a benzene ring with two chlorine atoms), so the value for this feature would be three. If there were multiple possible values of *k*, due to multiple common motifs, we used the maximum *k*.

As a third feature ('struct_similarity'), we calculated the average structural similarity score for the compounds *C*_*i*_, using the Tanimoto similarity method described (Figure 3), where the Tanimoto coefficient is calculated from the 554-substructure PubChem fingerprints.

#### Co-inhibiting 'secondary compound' fitness defect scores (ten features)

We identified the top ten heterozygous co-inhibiting compounds with *c*_*j*_, the compound of interest. We then used the fitness defect scores of these ten 'secondary compounds' for the gene of interest, *g*_*i*_, as ten features ('secondary_ligands'). Our hypothesis is that co-inhibiting compounds share physical targets, suggesting that if the instance compound targets the instance protein, inhibiting the heterozygous deletion strain, then co-inhibiting compounds would be more likely to inhibit the strain as well.

#### Co-inhibiting 'secondary compound' fitness defect scores: summary statistics (two features)

We summarized the top ten co-inhibiting heterozygous secondary compounds' fitness defect scores as their mean ('secondary_mean') and median ('secondary_median').

### Classification of drug targets

We applied several machine learning algorithms using the Weka packages [80]: Random Forest, Naïve Bayes, Decision Stump, Logistic Regression, Support Vector Machine (SVM: SMO algorithm), Decision Tree (J48 algorithm), and Bayesian Network. We tested their accuracy on the training sets using tenfold cross-validation (Supplementary Figure 7 in Additional file 1); Random Forest exhibited the greatest performance and was selected for future use. This algorithm constructs a 'random forest' of ten decision trees, each of which considers sqrt(M) + 1 random features, where M is the number of original features (20 in the case of considering all features, 1 in the case of considering fitness defect alone). The algorithm's decision is the mode of the decisions of the ten trees.

### Experimental testing of predicted drug-target interaction by overexpression

We filtered the top predicted interactions for further testing using the following criteria: the gene was essential or showed a fitness defect as a homozygous deletion strain in the absence of compound; the confidence value of predicted interaction (from the Random Forest algorithm) was ≥0.7 out of 1, high fitness defect (log ratio ≥ 5); the compound was not a frequently predicted interactor; and the protein and compound were reciprocal top ten sensitivity hits of each other. This yielded 12 pairs (Supplementary Table 1 in Additional file 1), four for which we were able to obtain overexpression plasmids and compound reagents: Cox17 with clozapine, Exo84 with nocodazole, Pop1 with nystatin, and Arc18 with nystatin.

#### COX17 overexpression

To create the overexpression strains, yeast strain Y258 (*MAT***a ***pep4-3, his4-580, ura 3-53, leu2-3,112 *[81]) was transformed with plasmids BG1805 (Open Biosystems, Huntsville, AL, USA): the vector is derived from pRSAB1234 from Erin O'Shea using sequence verified, *de novo *synthesized yeast or human *COX17 *under control of the Gal1-promoter. The BG1805 plasmid host was constructed as follows: BG1805 plasmid containing the *MTF1 *ORF was extracted from the Y258 yeast strain, amplified in DH5α *Escherichia coli*, purified, and restriction digested with *Bsr*GI. The plasmid lacking the gene insert was gel purified and recircularized by ligation with T4 ligase. This 'empty' expression vector was used as the control in our experiments, and was also used for subcloning both yeast and human Cox17, which where synthesized and sequence-verified by BioBasic (Markham, Ontario, Canada). The sequences for generating the gene inserts are shown in Additional file 1. PCR products and the empty vector BG1805 were co-transformed into Y258.

In the overexpression experiments, the strains were grown overnight at 30°C on selective, synthetic defined medium lacking uracil, supplemented with 2% raffinose [82]. The next day, cells were diluted to OD_595 _0.0625 in YP medium containing 2% galactose and 1% raffinose [82]. After 4 hours of growth, clozapine (dissolved in DMSO) from Tocris Bioscience (Ellisville, MO, USA) was added to a final concentration of 400 μM to the experimental wells. A corresponding amount of DMSO (2%) was added to the control wells. The plate containing the cells was inserted in a Tecan GENios microplate reader (Tecan Systems Inc., San Jose, CA, USA) at 30°C with orbital shaking. Optical density measurements (OD_595_) were taken every 15 minutes for 45 hours. Due to the non-linearity between OD and cell number at higher cell densities, the measured Tecan ODs were converted to 'real' ODs using the calibration function 'real OD' = -1.0543 + 12.2716 × measured OD.

#### Exo84, Pop1, and Arc18 overexpression

2u plasmids (backbone p5476; kind gift from Charlie Boone's laboratory) containing full-length ORFs *EXO84*, *POP1*, or *ARC18 *under their native promoter along with approximately 900 bp upstream and approximately 250 bp downstream of the coding region, and the KanMX cassette, were amplified in DH5α *E. coli *and used to transform the BY4743 wild-type strain (*MATa/α his3Δ 1/his3Δ 1 leu2Δ 0/leu2Δ 0 lys2Δ 0/LYS2 MET15/met15Δ 0 ura3Δ 0/ura3Δ 0*) lacking the KanMX-marker [71]. In the overexpression experiments, the strains were grown overnight at 30°C on rich medium (YPD) supplemented with 200 μg/ml G418 [82]. The next day, cells were diluted to OD_595 _0.0625 in fresh YPD) supplemented with 200 μg/ml G418, and drug or vehicle (2% DMSO) was added. Potential rescue by overexpression was tested using a range of drug concentrations: *EXO84 *was probed using 20 to 27 μM nocodazole, and *POP1 *and *ARC18 *using 700 to 1,300 nM nystatin. To monitor growth, a Tecan GENios microplate reader (Tecan Systems Inc.) was used at 30°C with orbital shaking. OD measurements (OD_595_) were taken every 15 minutes for 34 hours. Due to the non-linearity between OD and cell number at higher cell densities, the Tecan ODs were converted to 'real' ODs using the calibration function 'real OD' = -1.0543 + 12.2716 × measured OD.

## Abbreviations

ATC: Anatomical Therapeutic Chemical; COX: cytochome C oxidase; DMSO: dimethyl sulfoxide; FDA: Food and Drug Administration; HIP: haploinsufficiency profiling; HOP: homozygous profiling; IDF: inverse document frequency; OD: optical density; ORF: open reading frame; PDR: pleiotropic drug resistance; SMARTS: SMiles ARbitrary Target Specification; SMILES: Simplified Molecular Input Line Entry System; WHO: World Health Organization.

## Authors' contributions

MEH, RWD, DK, CN, and GG conceived of and designed the studies. MEH carried out the data analysis and drafted the manuscript. EE carried out the overexpression experiments. MEH, EE, DK, CN, GG, participated in manuscript improvements. All authors read and approved the final manuscript.

## Supplementary Material

Additional file 1 Supplementary Table 1, Supplementary Figures 1 to 10, and supplementary information. Click here for file
