# Supplementary material for: Systematic analysis of genome-wide fitness data in yeast reveals novel gene function and drug action
Source: Genome Biol. 2010 Mar 12;11(3):R30. doi: 10.1186/gb-2010-11-3-r30 (PMC2864570; doi:10.1186/gb-2010-11-3-r30)
Supplement: Additional file 1 — Supplementary Table 1, Supplementary Figures 1 to 10, and supplementary information. [file gb-2010-11-3-r30-S1.pdf]

Supplementary Information
Table of Contents

Hillenmeyer et al.
Systematic analysis of genome-wide fitness data in yeast reveals novel gene function and drug action

Page

1. Supplementary tables and figures …………………………….. 2

2. Supplementary table and figure legends ……………………… 13

3. Supplementary data …………………………………………… 16

4. Sequences used for generating the gene inserts ………………. 18

Supp Table 1

gene	drug	Tested for overexpression in this study	Confirmed by overexpression in this study	Confirmed by previous study
RPO31	cinerubin	no		
COX17	clozapine	yes	yes	
GLC7	calyculin a	no		Hoon et al., Nat Chem Biol, 2008
POP1	nystatin	yes	no	
GLC7	cantharidin disodium	no		McCluskey et al., Bioorganic & Medicinal Chemistry Letters, 2002
PDR5	econazole nitrate	no		Chen, J Bacteriology, 2001
ARP9	calyculin a	no		
YOL050C	idarubicin	no		
SQT1	cuso4	no		
ARC18	nystatin	yes	no	
EXO84	nocodazole	yes	yes	
GFA1	phenanthroline monohydrochloride	no		

Supp Fig 1

a

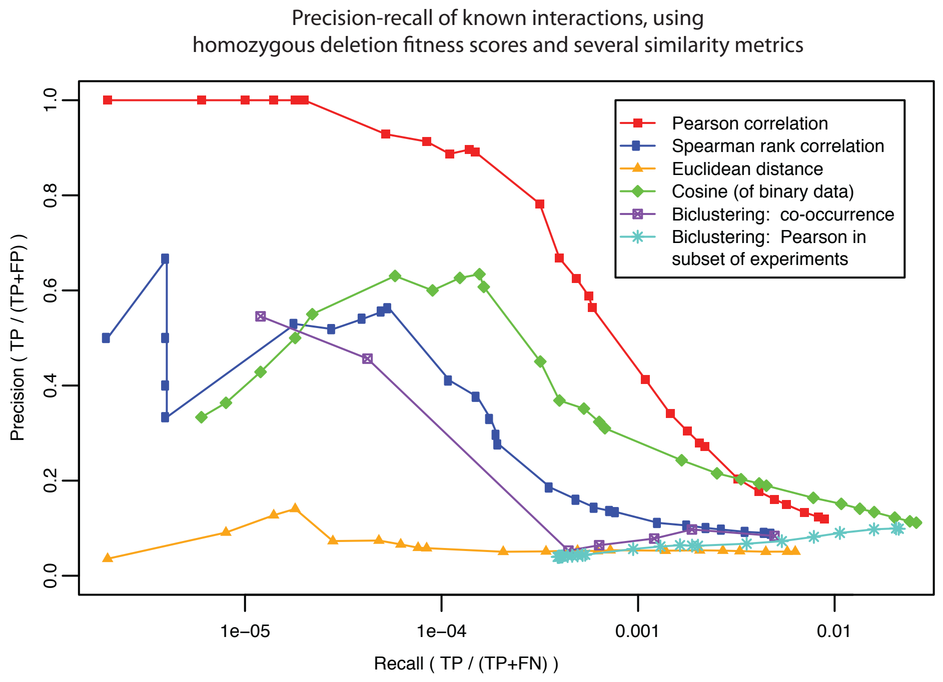

b

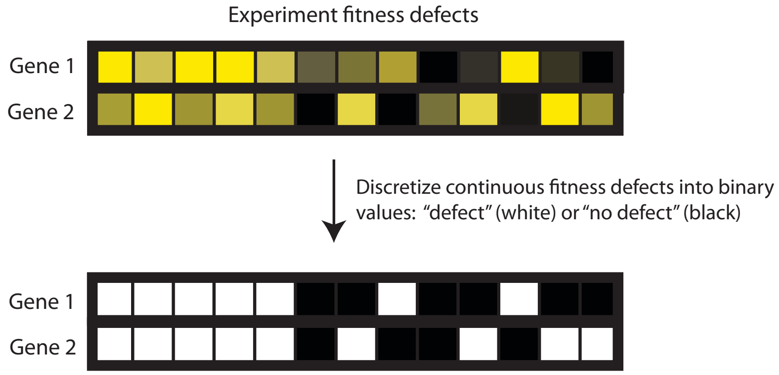

c

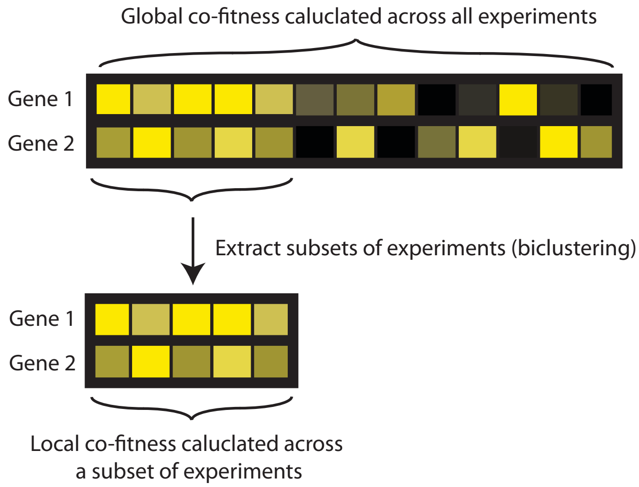

Supp Fig 2

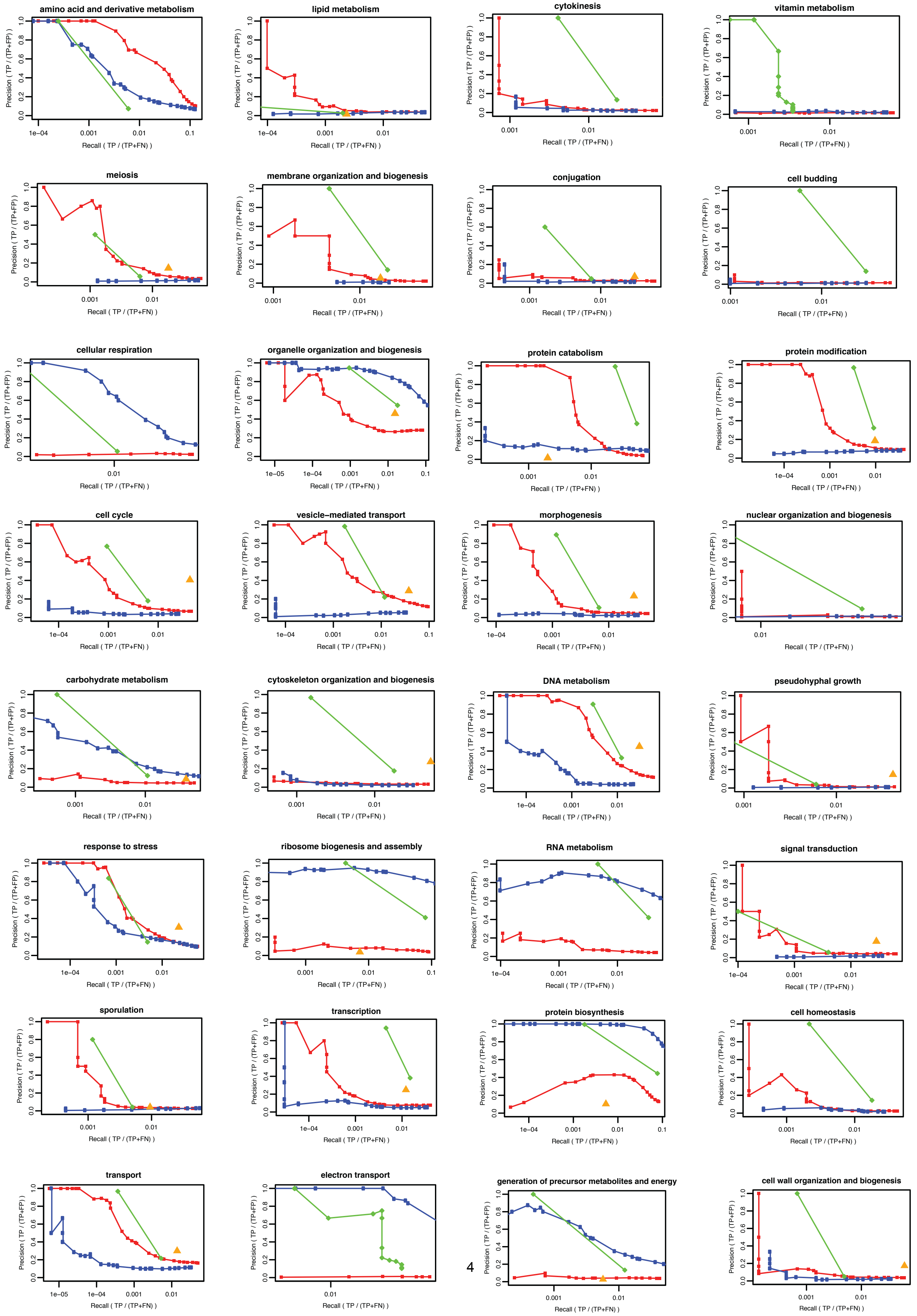
amino acid and derivative metabolism
lipid metabolism
cytokinesis
vitamin metabolism
meiosis
membrane organization and biogenesis
conjugation
cell budding
cellular respiration
organelle organization and biogenesis
protein catabolism
protein modification
cell cycle
vesicle–mediated transport
morphogenesis
nuclear organization and biogenesis
carbohydrate metabolism
cytoskeleton organization and biogenesis
DNA metabolism
pseudohyphal growth
response to stress
ribosome biogenesis and assembly
RNA metabolism
signal transduction
sporulation
transcription
protein biosynthesis
cell homeostasis
transport
electron transport
generation of precursor metabolites and energy
cell wall organization and biogenesis
Precision (TP / (TP+FP))
Recall (TP / (TP+FN))

Supp Fig 3

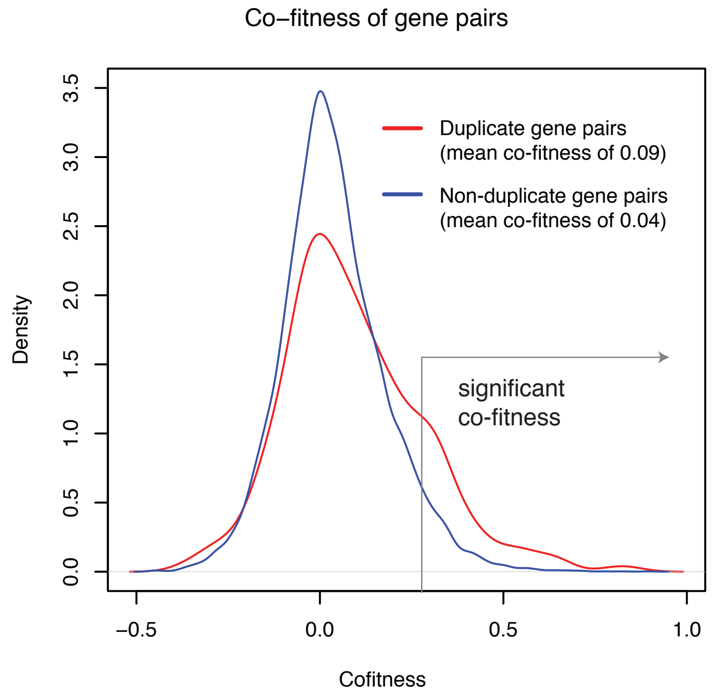
Co–fitness of gene pairs
Duplicate gene pairs
(mean co-fitness of 0.09)
Non-duplicate gene pairs
(mean co-fitness of 0.04)
significant
co-fitness
Density
0.0
0.5
1.0
1.5
2.0
2.5
3.0
3.5
–0.5
0.0
0.5
1.0
Cofitness

Supp Fig 4

a. Sequence similarity vs. heterozygous co-fitness

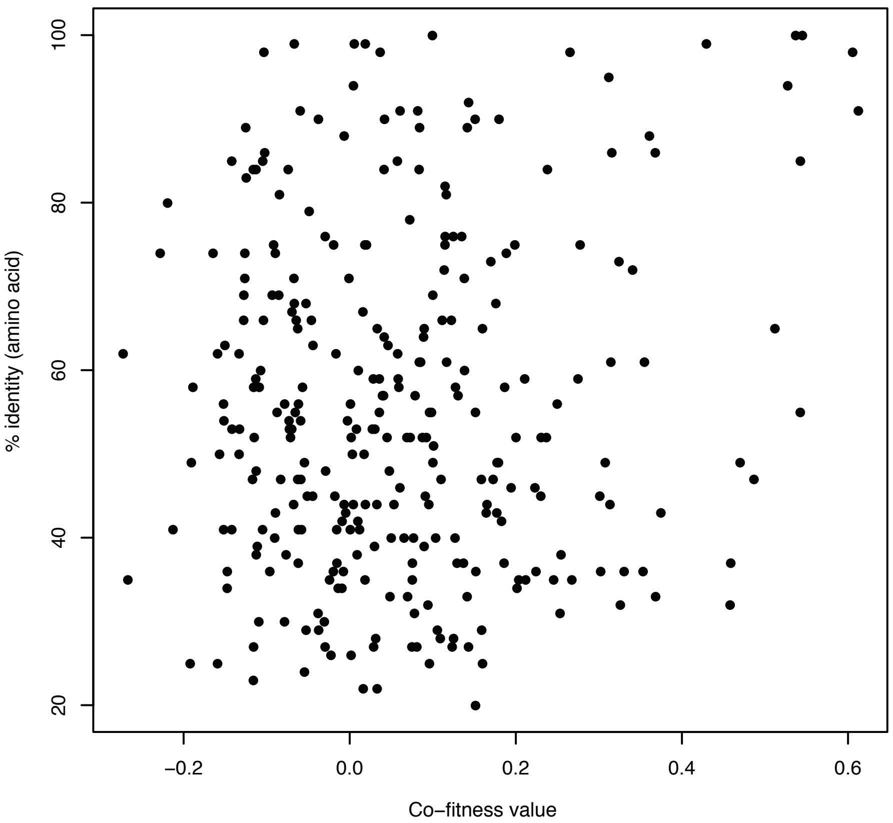

b. Sequence similarity vs. homozygous co-fitness

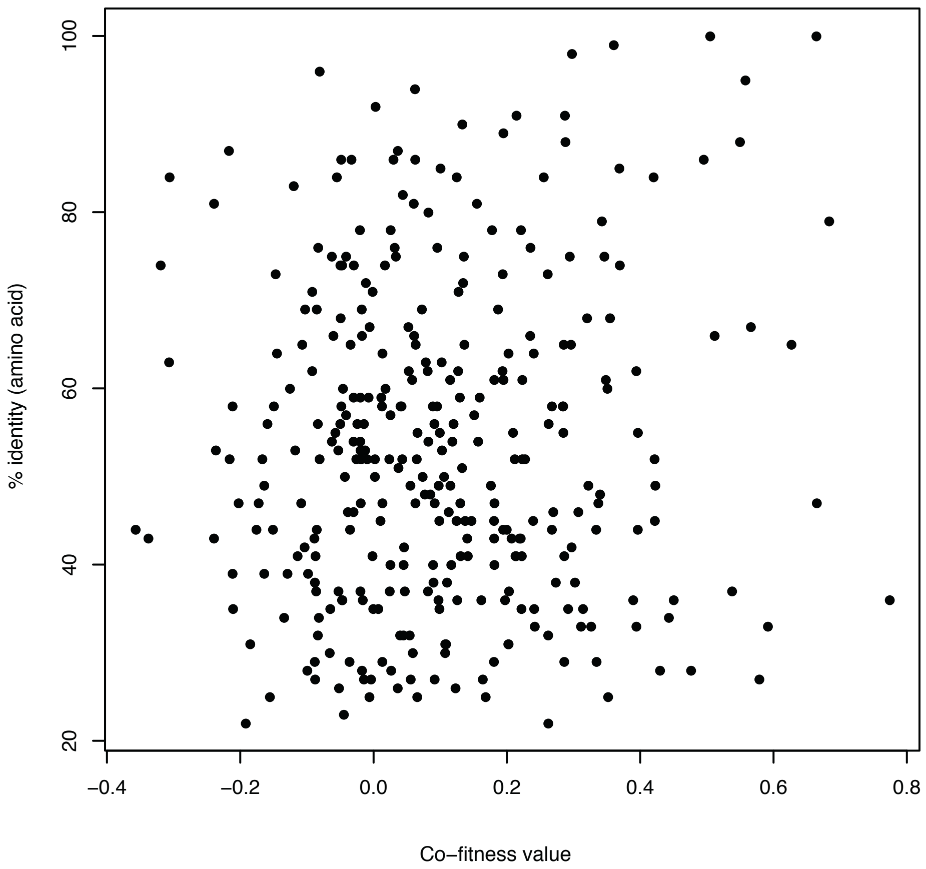

Supp Fig 5

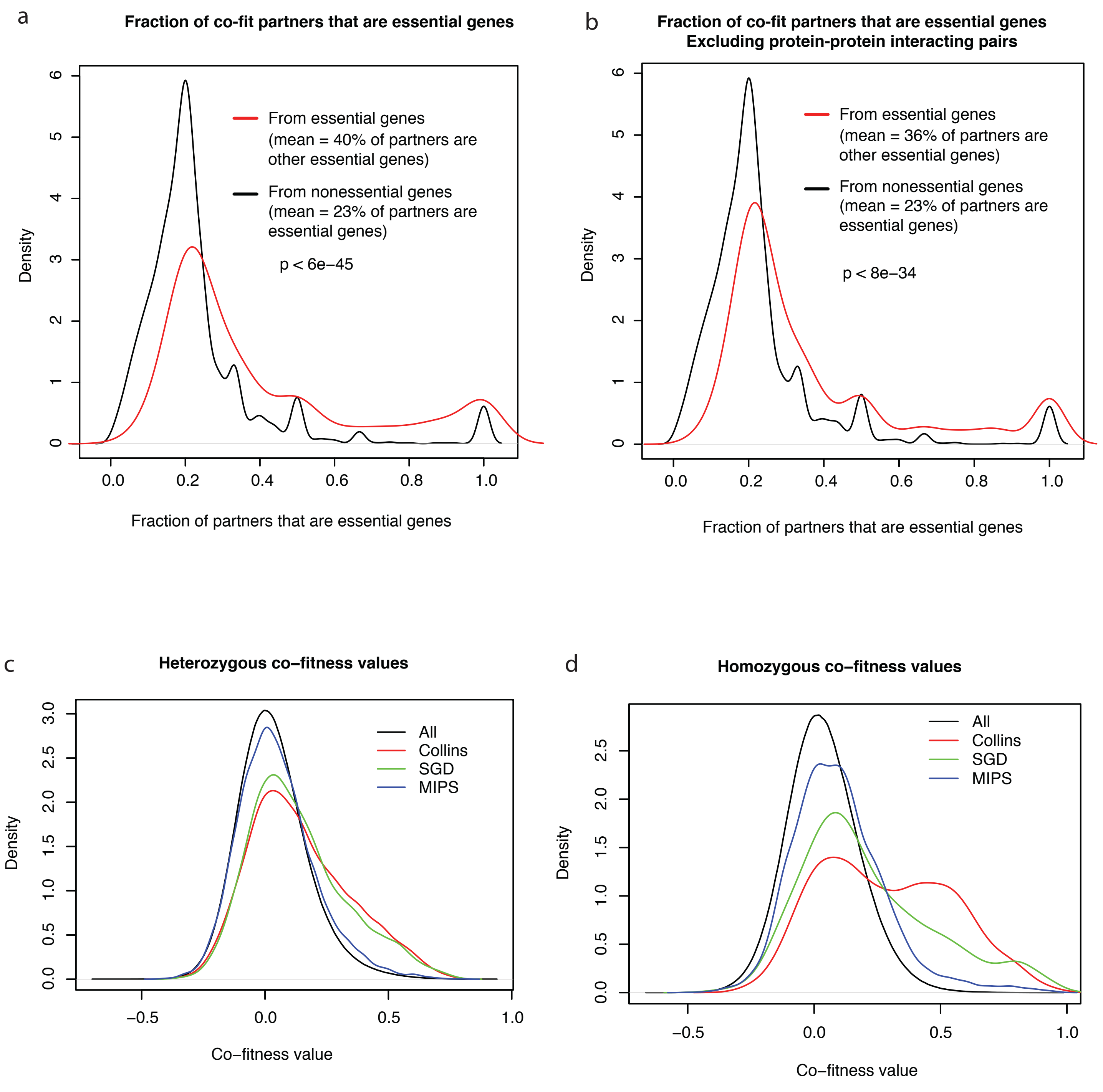
a
Fraction of co-fit partners that are essential genes
From essential genes
(mean = 40% of partners are
other essential genes)
From nonessential genes
(mean = 23% of partners are
essential genes)
p < 6e−45
Density
Fraction of partners that are essential genes
b
Fraction of co-fit partners that are essential genes
Excluding protein-protein interacting pairs
From essential genes
(mean = 36% of partners are
other essential genes)
From nonessential genes
(mean = 23% of partners are
essential genes)
p < 8e−34
Density
Fraction of partners that are essential genes
c
Heterozygous co−fitness values
All
Collins
SGD
MIPS
Density
Co−fitness value
d
Homozygous co−fitness values
All
Collins
SGD
MIPS
Density
Co−fitness value

Supp Fig 6

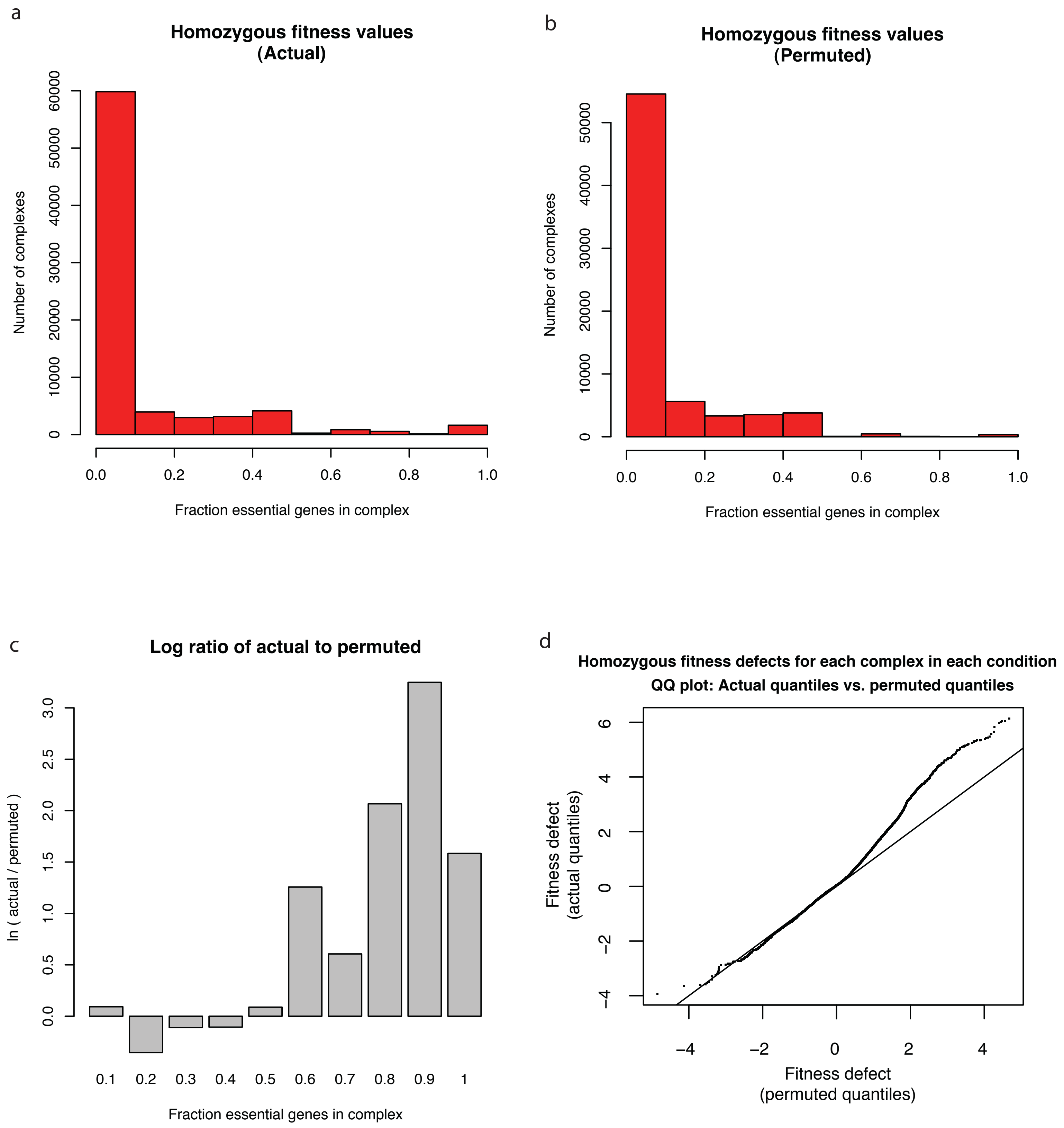

a
Homozygous fitness values
(Actual)
Number of complexes
0
10000
20000
30000
40000
50000
60000
0.0
0.2
0.4
0.6
0.8
1.0
Fraction essential genes in complex
b
Homozygous fitness values
(Permuted)
Number of complexes
0
10000
20000
30000
40000
50000
0.0
0.2
0.4
0.6
0.8
1.0
Fraction essential genes in complex
c
Log ratio of actual to permuted
ln (actual / permuted)
0.0
0.5
1.0
1.5
2.0
2.5
3.0
0.1
0.2
0.3
0.4
0.5
0.6
0.7
0.8
0.9
1
Fraction essential genes in complex
d
Homozygous fitness defects for each complex in each condition
QQ plot: Actual quantiles vs. permuted quantiles
Fitness defect
(actual quantiles)
−4
−2
0
2
4
6
−4
−2
0
2
4
Fitness defect
(permuted quantiles)

Supp Fig 7

a. Test set: Human homologs

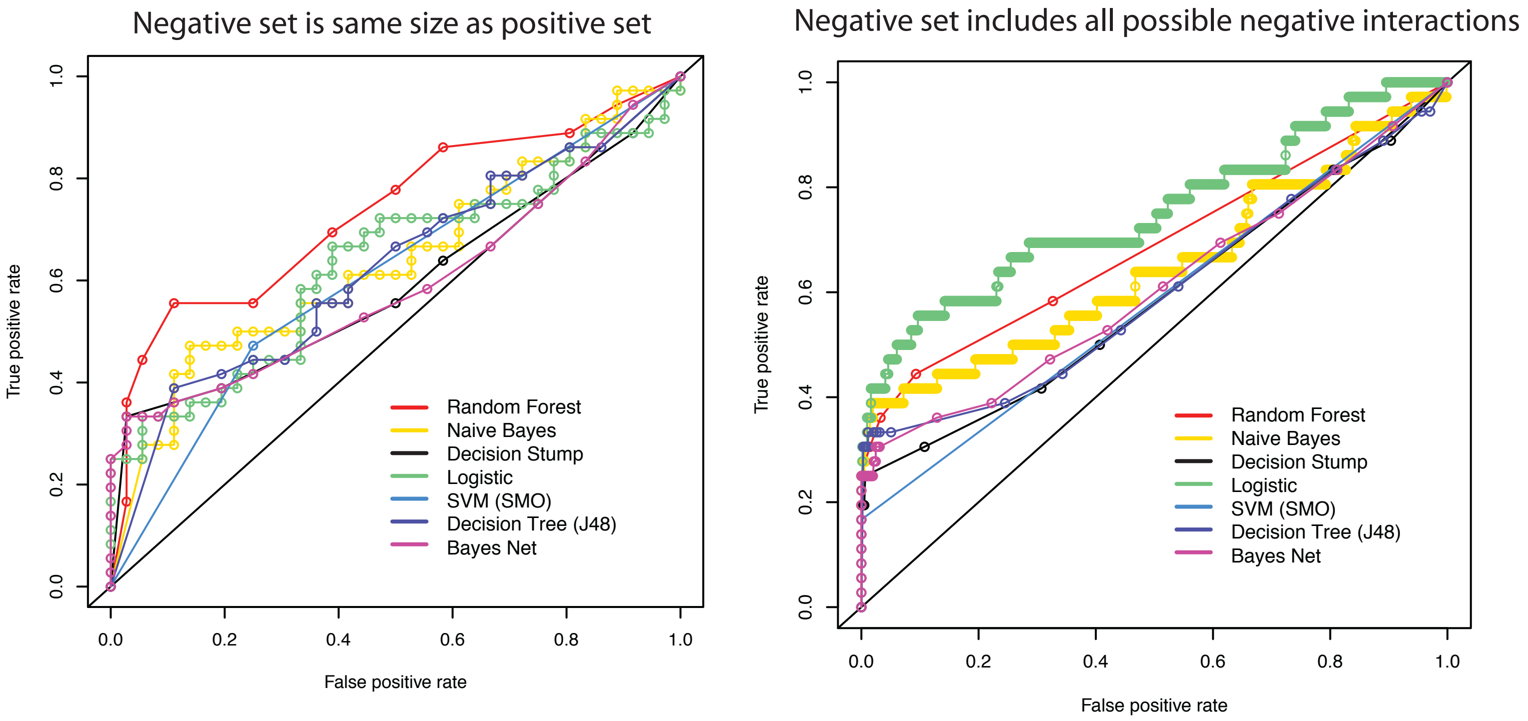

b. Test set: Yeast

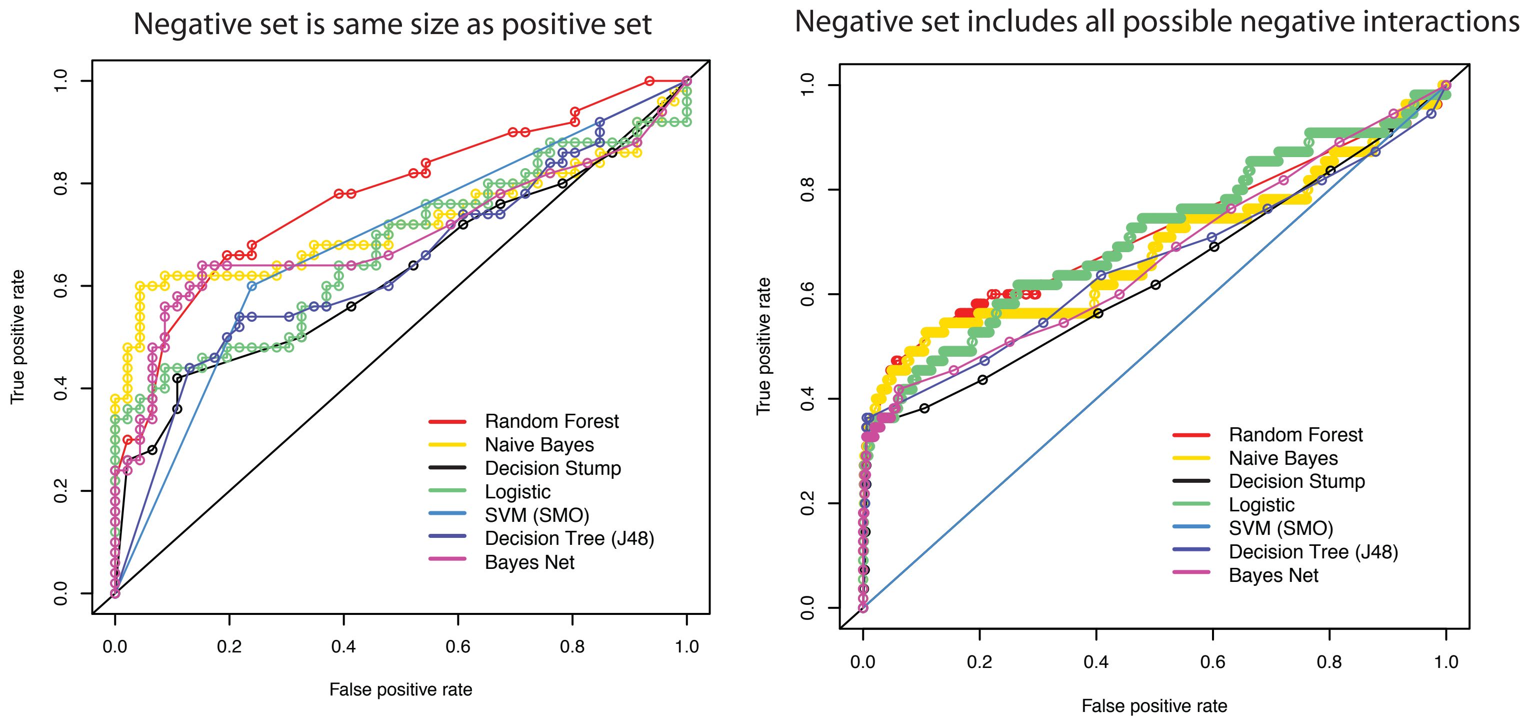

Supp Fig 8

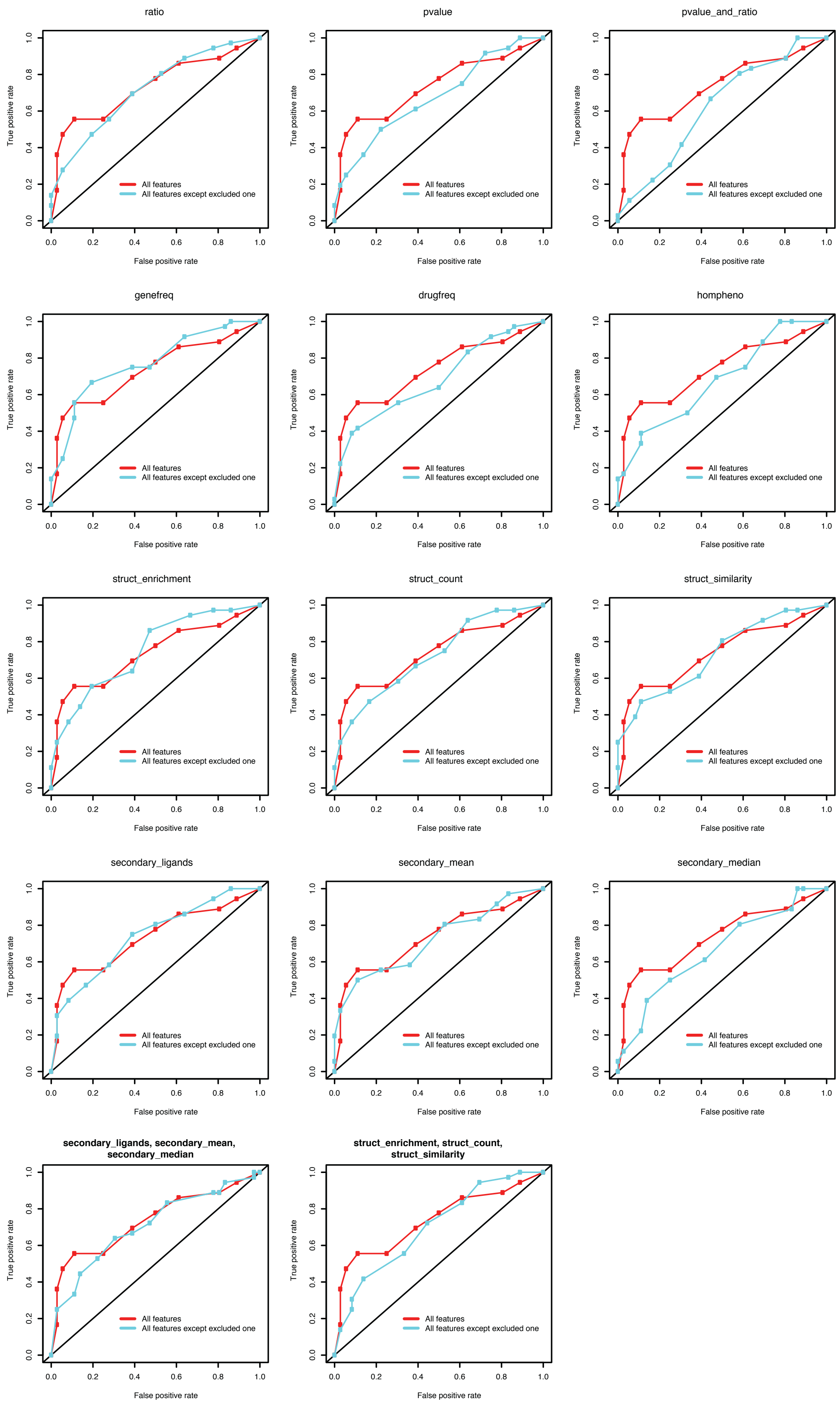
ratio
pvalue
pvalue_and_ratio
genefreq
drugfreq
hompheno
struct_enrichment
struct_count
struct_similarity
secondary_ligands
secondary_mean
secondary_median
secondary_ligands, secondary_mean, secondary_median
struct_enrichment, struct_count, struct_similarity
True positive rate
False positive rate
0.0
0.2
0.4
0.6
0.8
1.0
All features
All features except excluded one

Supp Fig 9

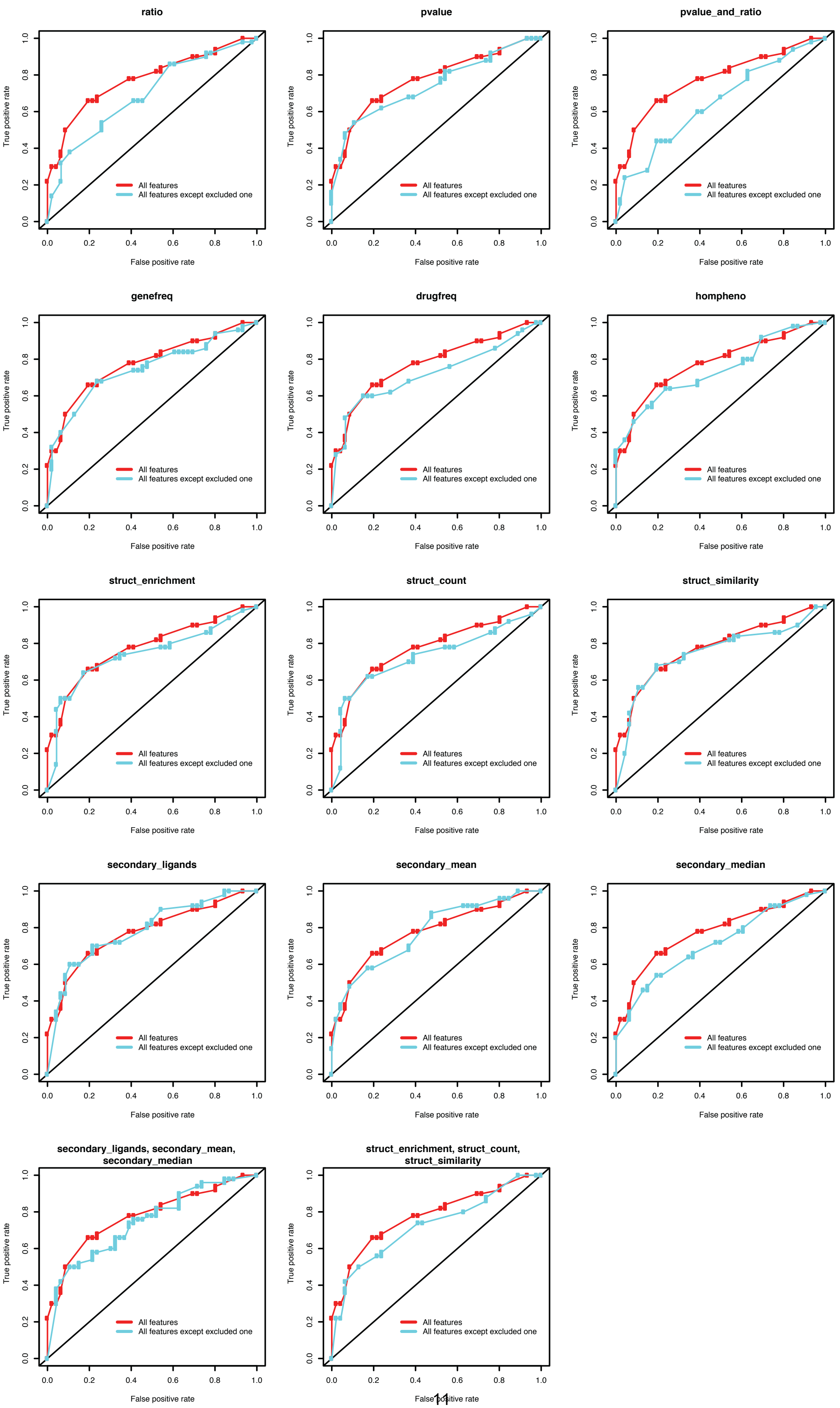
ratio
pvalue
pvalue_and_ratio
genefreq
drugfreq
hompheno
struct_enrichment
struct_count
struct_similarity
secondary_ligands
secondary_mean
secondary_median
secondary_ligands, secondary_mean, secondary_median
struct_enrichment, struct_count, struct_similarity
True positive rate
False positive rate
0.0
0.2
0.4
0.6
0.8
1.0
All features
All features except excluded one

Supp Fig 10

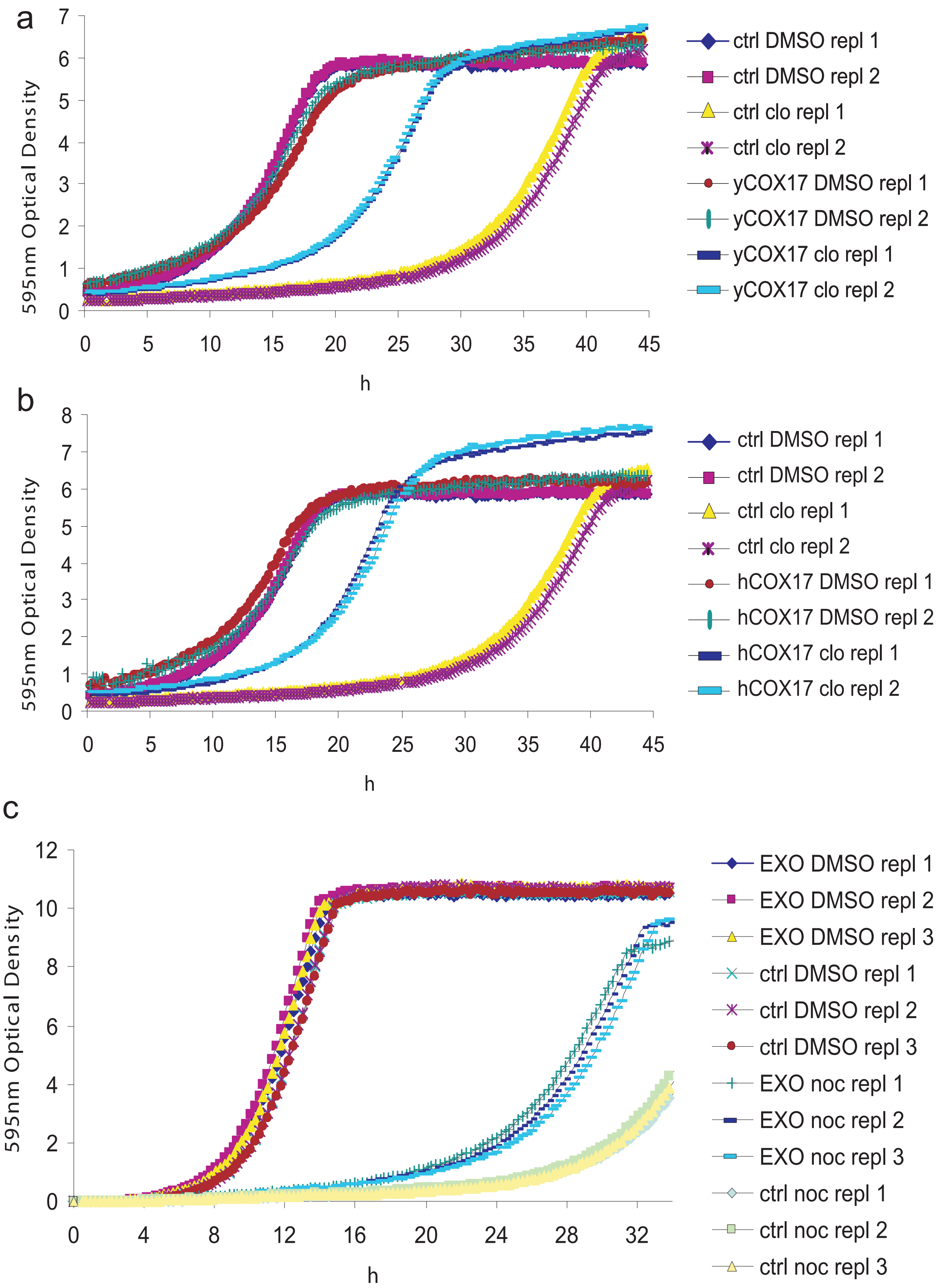
a
595nm Optical Density
h
ctrl DMSO repl 1
ctrl DMSO repl 2
ctrl clo repl 1
ctrl clo repl 2
yCOX17 DMSO repl 1
yCOX17 DMSO repl 2
yCOX17 clo repl 1
yCOX17 clo repl 2
b
595nm Optical Density
h
ctrl DMSO repl 1
ctrl DMSO repl 2
ctrl clo repl 1
ctrl clo repl 2
hCOX17 DMSO repl 1
hCOX17 DMSO repl 2
hCOX17 clo repl 1
hCOX17 clo repl 2
c
595nm Optical Density
h
EXO DMSO repl 1
EXO DMSO repl 2
EXO DMSO repl 3
ctrl DMSO repl 1
ctrl DMSO repl 2
ctrl DMSO repl 3
EXO noc repl 1
EXO noc repl 2
EXO noc repl 3
ctrl noc repl 1
ctrl noc repl 2
ctrl noc repl 3

2. Supplementary table and figure legends

Supplementary Table 1
The top 12 predicted interactions, as described in Materials and Methods. We were able to obtain overexpression plasmids and compound reagents for four of these pairs: Cox17 with clozapine, Exo84 with nocodazole, Pop1 with nystatin, and Arc18 with nystatin. We found evidence for two of these interactions (Cox17 with clozapine and Exo84 with nocodazole) in our overexpression study, and two more have been validated in previous studies (Glc7 with calyculin a [23], Glc7 with cantharidin [52], and Pdr5 with econazole [51]). Two of our tested interactions (Pop1 with nystatin and Arc18 with nystatin) were not supported by our overexpression study, but this was not surprising as nystatin is a membrane disruptor and individual rescue by a single target is unlikely.

Supplementary Figure 1
(a) Precision-recall curve for homozygous co-fitness, tested against a gold standard of ~500,000 functional interactions [13]. Six similarity metrics are shown. Pearson correlation across all experiments shows the greatest prediction performance.
(b) The real-valued fitness defect scores can be discretized according to an arbitrary threshold, converting the values into binary values of "fitness defect" (white) or "no fitness defect" (black). The threshold was determined by significant fitness defect ($p < 0.05$).
(c) Global similarity is calculated across all experiments. Biclustering is calculated using local similarity across a subset of experiments.

Supplementary Figure 2
Precision-recall curves for all 32 GO Slim categories for each of four high-throughput datasets co-fitness (red), co-expression (blue), synthetic lethality (yellow), and physical interaction (green). The curves illustrate the accuracy of each dataset to predict an expert-curated reference set of interactions. The optimal dataset has both high precision and high coverage (i.e. a point in the upper right corner). TP is the number of true positive interactions captured by the dataset; FP is the number of false positive interactions. Synthetic lethality networks have only one value for precision and coverage, because their links are binary, but correlation-based networks such as co-fitness and co-expression use an adjustable correlation threshold to define interactions: each point on the line corresponds to one threshold.

Supplementary Figure 3
Co-fitness of duplicated gene pairs (red) (i.e., a duplicated gene and its partner) and non-duplicated gene pairs (blue) in the homozygous dataset. Duplicated genes are more co-fit with their duplicated partners than are non-duplicated genes.

Supplementary Figure 4
(a) Sequence similarity (% identity of full length amino acid sequence) compared with heterozygous co-fitness value. Each point represents a pair of duplicated genes, whose names are shown to allow visual inspection.
(b) Sequence similarity (% identity of full length amino acid sequence) compared with

homozygous co-fitness value.

Supplementary Figure 5
(a) Fraction of heterozygous co-fit partners that are essential genes. To define a set of co-fit partners, we calculated a significant ($p < 0.01$) co-fitness threshold as > 0.47; any gene pairs having co-fitness greater than this threshold were defined as co-fit partners. For essential genes (red), 40% of co-fit partners on average were other essential genes. For non-essential genes (black), 23% of co-fit partners on average were other essential genes, a significant difference ($p < 6e-45$). This suggests that essential genes preferentially share phenotype with other essential genes.

(b) As in (a), but here we excluded any pairs that existed in any of the three protein interaction datasets discussed in the methods (MIPS, Collins, or SGD complexes). The difference remained significant ($p < 8e-34$), though slightly diminished. This suggests that the observed essential-essential relationships observed in (a) could not be attributed to essential protein complexes only; there are other essential systems (or perhaps undiscovered complexes) that act together.
(c,d) Distribution of (c) heterozygous, and (d) homozygous co-fitness for pairs of interacting proteins extracted from three datasets, compared with random pairs (black). "Collins" refers to [25], predicted pairwise protein interactions by combining data from two large-scale affinity precipitation studies [26-27]). "SGD" refers to GO component protein complexes downloaded from Saccharomyces Genome Database (ftp://genome-ftp.stanford.edu/pub/yeast/literature_curation/go_protein_complex_slim.tab), and complexes were converted to gene pairs by pairing each protein in a complex with every other protein in the complex (ignoring the fact that some proteins within a complex may not be directly interacting). "MIPS" refers to protein complexes downloaded from the Munich Information Center for Protein Sequences, with complexes converted to gene pairs by pairing each protein in a complex with every other protein in the complex.

Supplementary Figure 6
In rich medium, Hart *et al* [14] showed that, essential complexes were skewed towards being entirely essential or entirely nonessential Here we examined whether these findings hold for the conditions in our study. If the homozygous deletion strain caused a severe fitness defect ($p < 0.01$) in a particular condition, the gene was considered to be required for growth in that condition, and therefore is considered conditionally essential. We used the SGD complexes as described above and calculated the fraction of essential genes in each complex. 215 complexes (Materials and Methods) were considered separately for 418 conditions; the total number of test cases was 215 x 418 = 89,870.
(a) Histogram of the number of complexes having each fraction of essential genes (e.g., about 6000 complexes contained 0-10% essential genes).
(b) As in (a), but complexes were permuted as described in the Materials and Methods.
(c) Log ratio of (a) to (b). The fraction of complexes that were largely essential (right side of the histogram) was much greater for actual complexes than permuted complexes.
(d) The average fitness defect (as $\log_2$ ratio) of the complex, visualized as the quantiles (QQ plot) for the average fitness defect for actual complexes vs. permuted complexes. Actual complexes tended to have greater fitness defect, i.e., be "more" essential than

random complexes.

Supplementary Figure 7
Protein-ligand prediction accuracy of several algorithms on 10-fold cross validation. Each algorithm was provided with all features described in Materials and Methods. Negative examples were constructed as random interactions, as described in Materials and Methods, in both balanced (equal number of negative and positive examples) and unbalanced (all possible negative interactions) regimes.
(a) Performance within the test set of interactions involving human proteins that have yeast homologs.
(b) Performance within the test set of yeast interactions.

Supplementary Figure 8
Ablation analysis of features within the test set of interactions involving human proteins that have yeast homologs. The red line in each panel represents the Random Forests algorithm using all features, and the blue line in each panel represents the Random Forests algorithm using all features except the omitted one(s), denoted in each panel's title. Feature abbreviations are noted in their descriptions in Materials and Methods. The last two panels represent multiple omitted features, which are noted in the titles.

Supplementary Figure 9
Ablation analysis of features within the test set of yeast interactions. The lines are as described for Supplementary Figure 8.

Supplementary Figure 10
Reproducibility of the overexpression assays.
(a, b) Overexpression of Cox17 alleviates the sensitivity of the control to 400μM clozapine. The optical density at 595nm over time for Y258 cells harboring the a) yeast and b) human Cox17 overexpression construct compared to that of controls (ctrl) transformed with a plasmid lacking a gene insert.
(c) Overexpression of Exo84 alleviates the sensitivity of the control to 27uM nocodazole. The optical density at 595nm over time for BY4743 cells harboring the Exo84 overexpression construct compared to that of controls (ctrl) transformed with plasmid lacking a gene insert.
Replicate (repl) experiments are shown. For details, see Materials and Methods.

3. Supplementary Data

The supplementary data is available at:
http://chemogenomics.stanford.edu/supplements/cofitness

Explanation of files:

The analyses for this study were performed using data from Hillenmeyer et al., "The Chemical Genomic Portrait of Yeast: Uncovering a Phenotype for all Genes", Science 2008. Original fitness data is available for download from that paper's supplement.

Co-fitness of gene pairs

* Heterozygous deletion experiments: het.ratio_result_nm.goodbatch.cofitness.txt

Pearson correlation values for pairs of gene deletion strains across all heterozygous experiments in Hillenmeyer et al., Science 2008. The term "goodbatch" in the filename refers to the exclusion of problematic batches, as described in Hillenmeyer et al., Science 2008.

* Homozygous deletion experiments: hom.ratio_result_nm.cofitness.txt

Pearson correlation values for pairs of gene deletion strains across all homozygous experiments in Hillenmeyer et al., Science 2008.

Co-inhibition of chemical pairs

* Heterozygous deletion experiments: het.ratio_result_nm.goodbatch.coinhibition.txt

Pearson correlation values for pairs of heterozygous experiments across all gene deletion strains in Hillenmeyer et al., Science 2008. The term "goodbatch" in the filename refers to the exclusion of problematic batches, as described in Hillenmeyer et al., Science 2008.

* Homozygous deletion experiments: hom.ratio_result_nm.coinhibition.txt

Pearson correlation values for pairs of homozygous experiments across all gene deletion strains in Hillenmeyer et al., Science 2008.

Drug target prediction

We used two separate training sets:

1. Yeast: compound_target_training_set_yeast.txt

Training set of compound-target interactions from an expert-curated set of known interactions

2. DrugBank: compound_target_training_set_DrugBank.txt

Training set of compound-target interactions extrapolated from their homologs that existed in DrugBank.

Each training set was used to learn a Random Forest model of compound-target interactions, as described in the main text. The model was then applied to a test set comprising all possible interactions (all compounds with all heterozygous yeast strains). The following two files list the predicted confidence values (when the algorithm was able to make a prediction) for those test set interactions.

1. Yeast: compound_target_predictions_RandomForest_yeast.txt

2. DrugBank: compound_target_predictions_RandomForest_DrugBank.txt

The format of these files is a tab-delimited list of protein-compound interactions. Each row is an interaction, with columns:

* protein
* compound
* prediction score
* input features (fitness_defect_pvalue, fitness_defect_ratio, gene_sens_freq, drug_inhib_freq, hompheno, struct_enrichment, struct_count, struct_similarity, fitness_defect_pvalue_0, fitness_defect_pvalue_1, fitness_defect_pvalue_2, fitness_defect_pvalue_3, fitness_defect_pvalue_4, fitness_defect_pvalue_5, fitness_defect_pvalue_6, fitness_defect_pvalue_7, fitness_defect_pvalue_8, fitness_defect_pvalue_9, fitness_defect_pvalue_secondary_mean, fitness_defect_pvalue_secondary_median).
* compound prediction frequency (total number of predictions in which this compound appeared)

The input features are described more fully in the Materials and Methods in the main text.

Note that these prediction files include all possible interactions, not only the high-confidence ones. To filter the list to the highest-confidence interactions, we applied filters using the following criteria (described in the Materials and Methods in the main text): (1) the gene was essential or showed a fitness defect as a homozygous deletion strain in the absence of compound, (2) the confidence value of predicted interaction (from the Random Forest algorithm) was >= 0.7 out of 1, high fitness defect (log ratio >= 5), (3) the compound was not a frequently-predicted interactor (i.e., appeared in less than 1000

total predictions), and (4) the protein and compound were reciprocal top 10 sensitivity hits of each other, as determined by examination of the protein and compound in FitDB. This yielded 12 pairs (Supplementary Table 1).

4. Sequences used for generating the gene inserts

The sequences used were as follows (ORF in plain text, FLAG in *italics,* sequences complementary to *BsrGI* and *KpnI* sites in BG1805 in **bold):**

Yeast COX17-FLAG-Stop
COX17/YLL009C

ATGACTGAAACTGACAAGAAACAAGAACAAGAAAACCACGCGGAGTGCGAG
GACAAACCTAAGCCATGTTGCGTTTGTAAGCCAGAAAAGGAGGAGCGGGAT
ACATGCATCTTATTCAATGGACAAGACTCTGAAAAATGCAAGGAATTCATTG
AAAAGTACAAAGAGTGCATGAAGGGTTATGGCTTCGAAGTTCCAAGTGCAAA
T*GACTACAAGGATGACGACGATAAG*TGA

Human COX17-FLAG-Stop
niProtKB/Swiss-Prot: COX17_HUMAN, Q14061
ATGCCGGGTCTGGTTGACTCAAACCCTGCCCCGCCTGAGTCTCAGGAGAAGA
AGCCGCTGAAGCCCTGCTGCGCTTGCCCGGAGACCAAGAAGGCGCGCGATGC
GTGTATCATCGAGAAAGGAGAAGAACACTGTGGACATCTAATTGAGGCCCAC
AAGGAATGCATGAGAGCCCTAGGATTTAAAATA*GACTACAAGGATGACGACGA*
*TAAG*TGA

pUC57 containing yeast and human *COX17* was amplified in DH5α *E.coli* . The cloned constructs were PCR amplified with the following primers:
1. 5'-3' (forward) human Cox17:
GGAATTATCAACAAGTTTGTAATGCCGGGTCTGGTTGACTCAAAC

2. 5'-3' (forward) yeast Cox17:
GGAATTATCAACAAGTTTGTAATGACTGAAACTGACAAGAAACAA

3. 5'-3' (reverse) human and yeast Cox17:
ACTATAGGGGCGAATTGGGTA**TCACTTATCGTCGTCATCCTTGTA**
